# All-photonic quantum repeaters

**DOI:** 10.1038/ncomms7787

**Published:** 2015-04-15

**Authors:** Koji Azuma, Kiyoshi Tamaki, Hoi-Kwong Lo

**Affiliations:** 1 NTT Basic Research Laboratories, NTT Corporation, 3-1 Morinosato Wakamiya, Atsugi, Kanagawa 243-0198, Japan; 2 Center for Quantum Information and Quantum Control, Department of Physics and Department of Electrical and Computer Engineering, University of Toronto, Toronto, Ontario, Canada M5S 3G4

## Abstract

Quantum communication holds promise for unconditionally secure transmission of secret messages and faithful transfer of unknown quantum states. Photons appear to be the medium of choice for quantum communication. Owing to photon losses, robust quantum communication over long lossy channels requires quantum repeaters. It is widely believed that a necessary and highly demanding requirement for quantum repeaters is the existence of matter quantum memories. Here we show that such a requirement is, in fact, unnecessary by introducing the concept of all-photonic quantum repeaters based on flying qubits. In particular, we present a protocol based on photonic cluster-state machine guns and a loss-tolerant measurement equipped with local high-speed active feedforwards. We show that, with such all-photonic quantum repeaters, the communication efficiency scales polynomially with the channel distance. Our result paves a new route towards quantum repeaters with efficient single-photon sources rather than matter quantum memories.

## Abstract (web summary)

Quantum repeaters are needed for long-distance quantum communication but it is thought that they require matter quantum memories. Azuma *et al*. introduce an all-photonic quantum repeater based on flying qubits that scales polynomially with the channel distance without the need for matter quantum memories.

## Graphical abstract

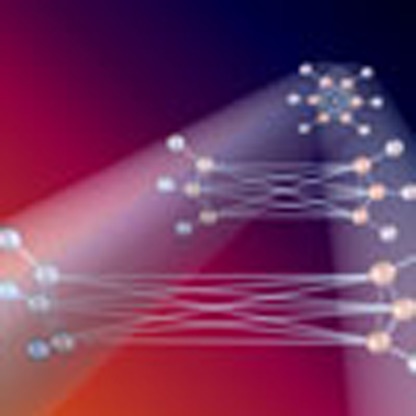

Quantum communication not only opens up opportunities for secure communication^1^,^2^ and the teleportation of quantum states^3^, but also is an important ingredient of the quantum internet^4^, which enables the distribution of entanglement over long distances. Since such a quantum internet will be useful for distributed quantum computing, distributed cryptographic protocols and dramatically lowering communication complexity, its realization is an important long-term scientific and technological goal. Thanks to the long coherence time of photons, photonic channels, for example, optical fibres, are often used for quantum communication. Nonetheless, owing to loss—which is the dominant noise^5^ for photons—the probability of successful transmission of a photon through an optical fibre decays exponentially. Consequently, the efficiency of this kind of quantum communication decreases exponentially with the communication distance, which is limited to hundreds of kilometres^6^.

To overcome such a distance limit, quantum repeaters^4^,^6^,^7^,^8^,^9^,^10^,^11^,^12^,^13^,^14^,^15^,^16^,^17^,^18^,^19^,^20^,^21^ that use repeater nodes between the sender (Alice) and the receiver (Bob) are needed to enjoy the polynomial scaling of the efficiency with the total distance. In contrast to conventional repeaters in classical communication, quantum repeaters cannot clone quantum signals^22^. Instead, as shown in Fig. 1a, the standard approach^4^,^6^,^7^,^8^,^9^,^10^,^11^,^12^,^13^,^14^,^15^,^16^,^17^,^18^,^19^ to quantum repeaters equips the repeater nodes with quantum memories, and starts with entanglement generation for the quantum memories between adjacent nodes via the transmission of photons entangled with the memories. Then, entanglement swapping^23^ is, one after another, performed at a node that has confirmed the existence of entanglement with other repeater nodes by receiving heralding signals from different repeater nodes at long distances. Thus, the quantum memories are at least required (i) to be entangled with photons (perhaps with a *telecom* wavelength for the fibre transmission) for the entanglement generation, and (ii) to be able to preserve entanglement faithfully at least until receiving the heralding signals for the entanglement swapping from the distant nodes. Without such quantum memories, the repeater protocols are inevitably reduced into quantum relay protocols^24^,^25^,^26^ with the exponential scaling (Fig. 1).

Earlier proposals^6^,^7^,^8^ for the realization regard an atomic ensemble as such a quantum memory with collectively enhanced coupling^8^ to photons for (i) and with infinite coherence time for (ii), relying on a *probabilistic* Bell measurement on single photons^27^. The protocols^6^,^7^,^8^ are simple in terms of the numbers of repeater nodes and the required matter quantum memories. However, unfortunately, if the coherence time of the matter quantum memories is *finite*—which is unavoidable as the dominant noise^5^ for matter—those simple protocols^6^,^7^,^8^ are shown^28^ to scale *exponentially* with (the square root of) the communication distance (irrespective of employed purification schemes^28^; Fig. 1). As seen from Fig. 1, the only solutions to overcome this problem would be (I) to boost the success probability of the Bell measurement (for example, by invoking a near-deterministic Bell measurement^29^ on single photons) or (II) to make the coherence time infinite by equipping the matter quantum memory with fault tolerance. But, either of these spoils the claimed simplicity of the original proposals^6^,^7^,^8^.

A solution for (I) or (II) may be to use matter *qubits* satisfying DiVincenzo's 2nd-to-5th criteria^30^ (initialization, quantum gates faster than decoherence time, universal gate set and readout) rather than the atomic ensembles, as in the protocols^9^,^11^,^12^,^13^,^14^,^15^,^16^,^17^,^18^,^19^,^20^,^21^. In fact, some of them work even with finite-coherence-time matter qubits^18^,^19^,^20^,^21^ (and also are fully fault tolerant^19^,^21^). In particular, Munro *et al*.^20^ have shown that matter qubits satisfying the criteria are no longer required to have a memory function for quantum repeaters—as expected from DiVincenzo's criteria^30^—if the coupling with photons is ultimately strong, faithful and efficient. As a result, their protocol achieves^20^ the highest repetition rate. However, unfortunately, matter qubits are normally less efficient^8^ in the coupling with photons for (i) than the atomic ensembles, and efficient coupling remains very challenging even with atomic ensembles despite recent experimental advances^4^,^5^,^6^,^31^,^32^. Thus, we have not yet been able to refute DiVincenzo's conjecture^30^ that the efficient coupling between a matter qubit and photons for (i)—corresponding to DiVincenzo's *extra* criterion^5^,^30^—is really hard. The only solution^18^ to compensate this inefficiency in entanglement generation under reasonable coherence time is to use a lot of matter qubits at each repeater node like the protocols^18^,^19^,^20^,^21^, satisfying even DiVincenzo's first criterion (scalability). However, this implies that the matter qubits in the quantum repeaters^9^,^11^,^14^,^15^,^16^,^17^,^18^,^19^,^20^,^21^ need to satisfy not only DiVincenzo's five (1st–5th) criteria^30^ for quantum computation but also his (really hard) extra criterion. Therefore, quantum repeaters^9^,^11^,^14^,^15^,^16^,^17^,^18^,^19^,^20^,^21^ may be more difficult than quantum computation. This is caused by the dogma^4^,^6^,^7^,^8^,^9^,^10^,^11^,^12^,^13^,^14^,^15^,^16^,^17^,^18^,^19^,^20^,^21^ of the requirements of matter quantum memories for quantum repeaters, which will remain undeniable without a future experimental breakthrough.

This paper disproves such a dogma that a demanding matter quantum memory is necessary for accomplishing quantum repeaters, by presenting *all-photonic* quantum repeaters. Our scheme uses only single-photon sources, linear optical elements, photon detectors, optical switches and a fast active feedforward technique (less than 150 ns (ref. 33)), similar to optical quantum computation^29^,^34^,^35^. However, our protocol is proven to be much easier than the quantum computation^29^,^34^,^35^, in contrast to the conventional quantum repeaters^4^,^6^,^7^,^8^,^9^,^10^,^11^,^12^,^13^,^14^,^15^,^16^,^17^,^18^,^19^,^20^,^21^. Moreover, the all-photonic nature of our repeaters has the following advantages that should be distinguished from ones of quantum repeaters based on matter quantum memories^4^,^6^,^7^,^8^,^9^,^10^,^11^,^12^,^13^,^14^,^15^,^16^,^17^,^18^,^19^,^20^,^21^. First, the heralding signals for the entanglement swapping are sent and received within the same repeater nodes, rather than between different repeater nodes at long distances, which reduces the transmission distance and time of the heralding signals to zero, in principle. This feature allows us to increase the repetition rate of our protocol as high as one wants within those of assumed photonic devices, comparable with speediest protocol by Munro *et al*.^20^ Second, even if we use a single-photon source based^36^ on a matter qubit, the matter qubit is no longer required to have a deterministic interaction with photons as well as to have long coherence time (and, of course, a matter quantum memory^6^,^7^,^8^,^31^ can be diverted to a single-photon source), let alone to satisfy all DiVincenzo's criteria^30^. Third, photonic quantum interfaces^37^,^38^ could be unnecessary. Finally, our protocol could work at room temperature.

## Results

### Main concept

We draw our protocol from a concept, ‘time reversal', underlying the distinguished findings in quantum information theory, such as measurement-based quantum computation^39^,^40^ and measurement-device-independent quantum key distribution (QKD)^41^. In fact, our protocol corresponds to the time reversal of the conventional quantum repeaters^4^,^6^,^7^,^8^,^9^,^10^,^11^,^12^,^13^,^14^,^15^,^16^,^17^,^18^,^19^, where entanglement swapping is performed before entanglement generation. This is an innovative part of our proposal. As an example to achieve such a time-reversed quantum repeater scheme, we use cluster-state^40^ flying qubits rather than simple Bell pairs, in contrast to existing quantum repeaters^4^,^6^,^7^,^8^,^9^,^10^,^11^,^12^,^13^,^14^,^15^,^16^,^17^,^18^,^19^. As our protocol is the time-reversed version of a conventional quantum repeater protocol with polynomial scaling, our protocol follows the same scaling. In what follows, we detail these in order.

### Conventional quantum repeaters

**Figure 1:**
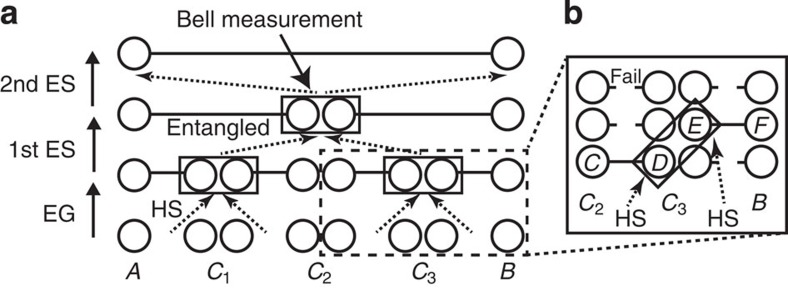
Quantum repeater protocol. (**a**) Conventional protocols^4^,^6^,^7^,^8^,^9^,^10^,^11^,^12^,^13^,^14^,^15^,^16^,^17^,^18^,^19^,^21^ and (**b**) parallel preparation of entangled pairs. (**a**) The repeater protocol is a way to supply entanglement with two-end parties, Alice (*A*) and Bob (*B*), by using repeater nodes {*C*_*i*_}_*i*=1,2,…,*n*_ (*n*=3 here). The protocol starts with entanglement generation (EG) through transmitting photons between adjacent repeater nodes, followed by recursive applications of the entanglement swapping (ES). The ES at a repeater node (for example, 2nd-round ES) starts only after the node receives signals for heralding the successful preparation of two entangled pairs at the previous round (1st-round ES). This preparation may be executed in a parallel manner as in (**b**) by using multiple quantum memories, where the heralding signals (HSs) are used to pick up an appropriate pair. Even in this way, if the EG and ES succeed only probabilistically as in protocols^4^,^6^,^7^,^8^,^10^,^15^, the total time for the transmission of the HSs alone is in the order of the communication time over the total distance *L*. As the matter qubits decay exponentially^5^ with this time, the protocols^4^,^6^,^7^,^8^,^15^ scale exponentially with the square root of the distance *L* between Alice and Bob irrespectively of the employed purification schemes^28^. If HSs are not exchanged, the protocol^4^,^6^,^7^,^8^,^10^,^15^ is merely the quantum relay^24^,^25^,^26^ with exponential scaling.

We start by considering the essential of the polynomial scaling of the conventional quantum repeaters (see Fig. 1), that is, the execution of the entanglement swapping upon confirming the existence of entangled pairs. Entanglement swapping is a way to share an entangled pair over a longer distance through connecting two (short) entangled pairs. Given an entangled state between systems *C* and *D* and an entangled state between systems *E* and *F* (Fig. 1b), it is possible to establish entanglement between systems *C* and *F*, by performing the Bell measurement on the systems *D* and *E*. Hence, if distances between *CD* and between *EF* are *l* and if *DE* are held at a single node, the entanglement swapping presents an entangled pair *CF* separated by distance 2*l*.

If we regard this entanglement swapping as the one implemented in a round (Fig. 1b) of a quantum repeater protocol (Fig. 1a), the entangled pairs *CD* and *EF* correspond to those prepared through the success of all the relevant entanglement generation processes and all the previous rounds of entanglement swapping. These entanglement preparations can be repeatedly applied to the specific qubits *CD* and *EF* until they are successfully entangled, as in proposed protocols^6^,^7^,^8^,^15^. However, in this case, owing to the fact that the entanglement preparations for *CD* and for *EF* are independent and merely probabilistic processes, the timings of successfully producing the entangled pairs *CD* and *EF* are not necessarily the same, which would require additional memory time for waiting the joint success event.

Instead, we can use a parallel procedure (as in Fig. 1b) to synchronize the successes of the entanglement preparations. In this method, each of the entanglement preparations for *CD* and for *EF* is executed *in* parallel by applying it to a sufficiently large number of qubits in order to successfully produce at least one entangled pair. Then, the prepared entangled pairs to be referred to as *CD* and *EF* appear simultaneously. Although this method reduces the requirements for the memory time of qubits, it still requires the qubits to have long memory time. In fact, the node to perform the Bell measurement on the counterparts *DE* needs to wait for the arrivals of heralding signals for specifying the qubits *DE* among many candidates at the same node (as shown in Fig. 1b). Then, as inferred by Fig. 1a, we notice an inherent problem of the quantum communication: the heralding signals should travel over long distances. This transmission time is at least the classical communication time between adjacent repeater nodes, and can be extended to the order of the communication time over the total distance if the entanglement swapping works only probabilistically as in simple schemes^6^,^7^,^8^,^10^,^15^. Owing to this waiting time, the conventional quantum repeaters^4^,^6^,^7^,^8^,^9^,^10^,^11^,^12^,^13^,^14^,^15^,^16^,^17^,^18^,^19^,^21^ need memory time and the repetition rate is limited.

The long waiting time for the transmission of the heralding signals becomes a problem even for an all-photonic quantum repeater scheme because the waiting time corresponds to the losses for the photonic qubits. To overcome this problem, we introduce an all-photonic time-reversed version of the conventional quantum repeaters^4^,^6^,^7^,^8^,^9^,^10^,^11^,^12^,^13^,^14^,^15^,^16^,^17^,^18^,^19^, where the waiting time could be made zero in principle.

### All-photonic time-reversed quantum repeaters

**Figure 2:**
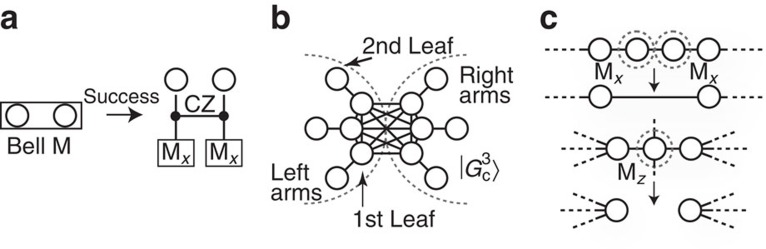
Linear-optics-based Bell measurement and cluster states. (**a**) Bell measurement based on linear optical elements and photon detectors^27^. If it succeeds, it works as the controlled-*Z* (CZ) gate followed by the *X*-basis measurements. If it fails, *Y*-basis measurements are applied for the existing photons, and, for lost photons, it informs us of the photon losses. (**b**) Complete-like cluster states 
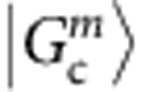
 (for the case of *m*=3). The state 
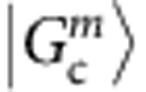
 has 2*m* arms, each of which is composed of 1st-leaf and 2nd-leaf qubits. The edge represents the past application of the CZ gate to qubits initialized in state 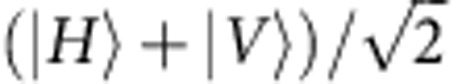, implying the existence of entanglement between them^40^. Here, |*H*〉 and |*V*〉 represent a basis of a single-photon qubit. The 1st-leaf qubits correspond to the memories held by a single repeater node in the conventional repeaters (for example, repeater node *C*_3_ in Fig. 1b). Single photons belonging to left arms (right arms) are to be sent to the left-hand-side (right-hand-side) adjacent receiver node (see Fig. 3). (**c**) Two adjacent *X*-basis measurements M_*X*_ on a linear cluster remove the qubits and directly connect their neighbours^40^. The *Z*-basis measurement M_*Z*_ on a qubit removes the qubit^40^.

Let us begin by specifying the role of the Bell measurement on the counterparts *DE* of the entangled pairs *CD* and *EF* in the parallel procedure of Fig. 1b. Here, the Bell measurement implicitly plays a role to entangle qubits *D* and *E* at a moment, as it can be regarded as an entangling operation followed by *X*-basis measurements (see Fig. 2a). Then, the time reversal of the whole process may be as follows: we first generate entanglement between *DE*, and then create entanglement between *CD* and between *EF*, which is followed by *X*-basis measurements on *DE*. However, at the beginning of this time-reversed protocol, it is impossible to specify the qubits *DE* among the many candidate qubits at the same node (as shown in Fig. 1b), because the heralding signals for the specification will be given after the successful entanglement preparations between *CD* and between *EF*. Thus, we propose to use the cluster state 
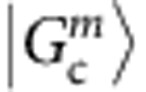
 that has 2*m* arms composed of 1st-leaf and 2nd-leaf qubits (see Fig. 2b). Here the 1st-leaf qubits serve as the candidate qubits at the same node and any pair of the 1st-leaf qubits is completely connected by edges that, respectively, represent the existence of entanglement. Then, since every pair of the 1st-leaf (candidate) qubits in the state 
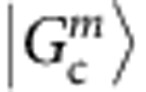
 is already entangled, in contrast to conventional repeater protocols^4^,^6^,^7^,^8^,^9^,^10^,^11^,^12^,^13^,^14^,^15^,^16^,^17^,^18^,^19^, we are not required to perform the (possibly probabilistic) Bell measurement on the qubits *DE*, let alone to specify the qubits *DE* in advance. Thus, the only remaining task at this point is to execute the *X*-basis measurements on the qubits *DE* according to the heralding signals that are to be given later.

**Figure 3:**
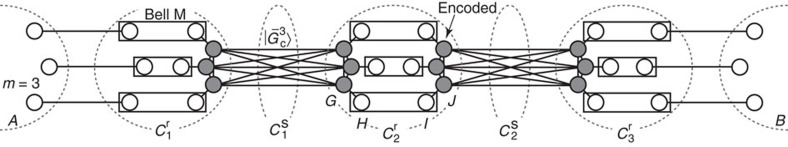
All-photonic quantum repeater protocol. The protocol is defined as follows: (i) Alice (Bob) prepares *m* single photons that are maximally entangled with her (his) local qubits and sends them to the adjacent receiver node 
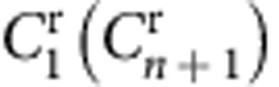
. At the same time, any other source node 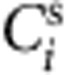 prepares the encoded complete-like cluster state 
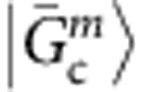
, and the left (right) arms are sent to the left-hand (right-hand) adjacent receiver node 
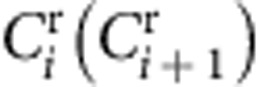
. (ii) On receiving the single photons (this moment in the case of (*m*, *n*)=(3, 2) is snapshot as an example), every receiver node applies the Bell measurement of Fig. 2a to the *m* pairs of the 2nd-leaf qubits of the left and right arms. (iii) If one of the Bell measurements succeeds, the receiver node performs the loss-tolerant *X*-basis measurements on the 1st-leaf qubits on the successful arms, and makes the loss-tolerant *Z*-basis measurements on all the other 1st-leaf qubits. If all the *m* Bell measurements or one of the loss-tolerant measurements on the 1st-leaf qubits fails, the receiver node regards this trial as failure. (iv) Finally, the receiver nodes announce all the measurement outcomes to Alice and Bob, and the protocol succeeds when no receiver node judges this trial as failure.

As we have seen, the 1st-leaf qubits of the state 
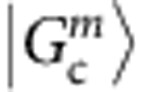
 correspond to quantum memories at a single repeater node in the conventional repeaters. In this analogy, the 2nd-leaf qubits serve as the single photons to supply entanglement to the 1st-leaf qubits between adjacent repeater nodes, that is, they are used for the entanglement generation process. To see this, let us consider a process to connect 1st-leaf qubits *G* and *J* in repeater node 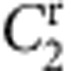 of Fig. 3. As the 1st-leaf qubits *G* and *J* are, respectively, entangled with the 2nd-leaf qubits *H* and *I*, if a linear-optics-based Bell measurement of Fig. 2a on the 2nd-leaf qubits *H* and *I* succeeds, the 1st-leaf qubits *G* and *J* are entangled, and they become the candidates for the qubits *DE* that are to receive the *X*-basis measurements. On the other hand, if the linear-optics-based Bell measurement fails owing to the photon losses of the 2nd-leaf qubits or the bunching effect of the photons, we apply *Z*-basis measurements on the 1st-leaf qubits *GJ*. These *Z*-basis measurements remove the corresponding arms without affecting the entanglement structure of the other arms of 
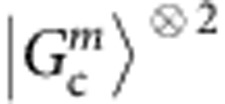
 according to the rule of Fig. 2c. This connection process for the 1st-leaf qubits can be executed in parallel for any arm of the state 
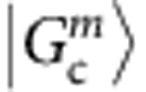
, which corresponds to the parallel entanglement generation (Fig. 1b) in the conventional quantum repeaters in Fig. 1a.

Note that the connection process requires the heralding signals from the 2nd-leaf qubits to the 1st-leaf qubits. If the 1st-leaf qubits were matter qubits that are stationary at a repeater node, the heralding signals would still be exchanged between adjacent repeater nodes, requiring the transmission time whose minimum ranges from hundred microseconds to milliseconds. Thus, the role of the 1st-leaf qubits could still be challenging for matter quantum memories from the current status^6^,^31^,^32^. However, in our proposal, the 1st-leaf qubits are composed of single-photon qubits. Thus, the 1st-leaf qubits can be sent with the 2nd-leaf qubits, which holds the transmission time of the heralding signals to the fundamental minimum, that is, the local active feedforward time. However, this causes an alternative problem that we need to apply single-qubit measurements on the the 1st-leaf qubits faithfully even under the photon losses as well as small errors of the transmission. But, as the transmission is performed merely between adjacent repeater nodes and the losses and the channel errors are thus independent of the total distance, they can be overcome by invoking a loss-tolerant scheme to execute a single-qubit measurement for the 1st-leaf qubits, say a protocol of Varnava *et al*.^34^ More specifically, instead of the state 
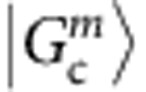
, we use its encoded version 
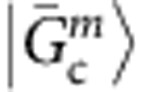
, that is, the complete-like cluster state 
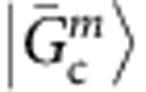
 with the encoded 1st-leaf qubits that are coloured in grey in Fig. 3.

The state 
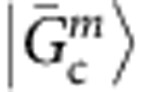
 can be generated locally using a preparation protocol of Varnava *et al*.^42^,^43^ This protocol synchronizes the generation of single photons, the application of the single-qubit and two-qubit linear-optics-based measurements and adaptive routing of single photons that succeeded as candidates for the final state 
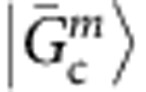
, which proceeds in a knockout tournament manner. This synchronized parallel procedure sacrifices a polynomial number of single photons for the number of qubits of 
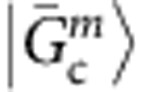
, but generates 
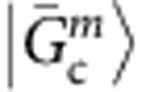
 in very-short constant time *τ*_c_ irrespective of photon loss. Such a small *τ*_c_ necessitates the use of fixed and predetermined optical delay lines such as optical fibres (but not variable buffers or memories). The detail is given in Supplementary Notes 1 and 2, which explicitly determines the preparation time *τ*_c_ that should be translated into the corresponding inherent loss probability for individuals of photons in state 
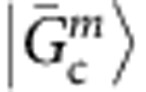
.

Conceptually, encoding for the state 
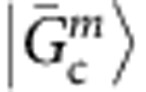
 by Varnava *et al*.^34^ is done by replacing a qubit being to receive a single-qubit measurement under loss with an encoded qubit composed of plural physical qubits. The loss-tolerant measurement is performed with an arbitrary high success probability via only single-qubit measurements on the physical qubits, as long as the loss probability for the physical qubits is less than 50% (corresponding to the loss of a 15-km optical fibre). Thus, in our protocol, the loss for the 1st-leaf qubits should be less than 50% by adjusting the transmission distance. This limitation corresponds to an analogy of the one on the quantum memory in the conventional quantum repeaters, although they differ^5^ in the types of dominant noises (loss and depolarization (or dephasing)).

In addition to the tolerance to the loss, as seen in Supplementary Note 1, remarkably, it turns out that the scheme of Varnava *et al*.^34^ allows us to perform *Z*-basis or *X*-basis measurement faithfully even under general errors. Thus, this scheme highly fits with our repeater scheme that needs (loss-tolerant) *Z*-basis and *X*-basis measurements only.

Notice, however, that scheme by Varnava *et al*. is less robust and loss-tolerant when non-Pauli measurements are performed. As universal optical quantum computing^29^,^34^,^35^ requires such non-Pauli measurements, the scheme by Varnava *et al*. requires more overhead and has a much lower error threshold in the case of universal optical quantum computing. This highlights the difference in the performance of scheme by Varnava *et al*. between the two applications—quantum repeaters and universal optical quantum computation.

To see how our protocol runs more precisely, we describe the whole protocol. In the repeater protocol, all the repeater nodes between Alice and Bob separated by distance *L* are classified into two sets: a set of source nodes 
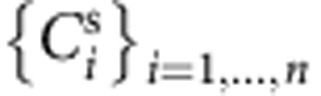
 and a set of receiver nodes 
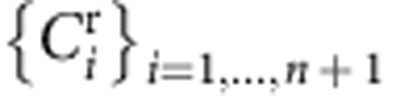
. The source nodes and the receiver nodes are placed alternatively and at regular intervals, and adjacent source nodes (adjacent receiver nodes) are separated, say *L*_0_=*L*/(*n*+1) apart. In addition, the arms of the state |*G*_c_^*m*^〉 are classified into the right-hand and left-hand sets as in Fig. 2b. Then, the repeater nodes run according to the protocol in Fig. 3.

### Applications

If we use our protocol in Fig. 3 for QKD, since Alice and Bob's raw key is virtually regarded as the one that is obtained by measurements on Alice and Bob's entangled pairs before starting our repeater protocol, Alice's and Bob's qubits in Fig. 3 can be virtual^41^. Moreover, as a possible correction to their qubits after the protocol is merely the application of a (unitary) Pauli operation, this correction corresponds to bit flips on their raw key. In addition, as every repeater node requires no classical communication with the other nodes according to the protocol of Fig. 3, the time required for each trial of the protocol is determined only by the number of the local active feedforwards used in steps (ii) and (iii) of the protocol. But this is merely one time (for the Bell measurements), because the loss-tolerant *X*-basis and *Z*-basis measurements in the step (iii) require no active feedforward (see Supplementary Note 1).

Without any need of quantum memories, our all-photonic quantum repeater scheme works not only in QKD, but also in many other quantum information processing protocols such as non-local measurements^44^,^45^ and cheating strategies^46^,^47^ in position-based quantum cryptography^48^. In those protocols, entanglement, once generated, is consumed immediately to generate classical output strings. For this reason, no quantum memory is needed in the protocol (see a flexibility of our repeater protocol in Supplementary Note 3). Furthermore, Pauli errors can be taken care of offline (that is, in the classical communication phase of the protocol).

For protocols that demand strictly a quantum output state, of course, quantum memories are needed. For instance, suppose Alice would like to transfer a quantum state to a distant observer, Bob via quantum teleportation^3^. Suppose further that Bob insists on keeping the final state as a quantum state (as he has no idea what measurement, if any, he might wish to perform in the future). In this case, the very fact that the final state is quantum means that the protocol requires effectively quantum memories with memory time in the order of classical communication time between Alice and Bob. However, even in this case, in contrast to the standard quantum repeaters^4^,^6^,^7^,^8^,^9^,^10^,^11^,^12^,^13^,^14^,^15^,^16^,^17^,^18^,^19^ as in Fig. 1, the memory time required in the quantum teleportation based on our repeater protocol scales only linearly with communication distance *L* like the speediest protocol^20^, differently from polynomial or subexponential scaling of the conventional ones^6^,^7^,^8^,^11^,^15^, which leads to greater suppression of the errors of the quantum memories (see the details in Supplementary Note 3).

### Scaling and performance

As expected from the time-reversed-like construction of our protocol itself, the average of the total photon number 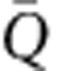 consumed in our protocol to produce an entangled pair between Alice and Bob scales only polynomially with the total distance. In addition, the average rate 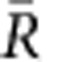 of our protocol to produce an entangled pair with a single repeater system is in the order of the repetition rate *f* of the slowest devices among single-photon sources, photon detectors, optical switches and active-feedforward techniques, which is in a striking contrast to the conventional repeaters^4^,^6^,^7^,^8^,^9^,^10^,^11^,^12^,^13^,^14^,^15^,^16^,^17^,^18^,^19^,^21^ whose rates are restricted by the communication time between adjacent nodes (from hundred microseconds to milliseconds), at least. The averaged fidelity 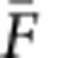 of the final entangled pair is degraded almost only by the small channel errors on the (bare) 2nd-leaf qubits contributing to the final pair, because the (encoded) 1st-leaf qubits enjoy the special robustness of the protocols of Varnava *et al*. for *Z*-basis and *X*-basis measurements. But, even these errors could be reduced if we could also instal entanglement purification like a protocol^16^ in our protocol in a time-reversed manner. The details are given in Supplementary Note 3 and Supplementary Discussion.

To show the scaling of our protocol explicitly, we present 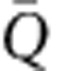, 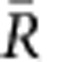 and the average fidelity 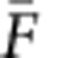 of the obtained entangled pair for two cases. Here we assume that photons always run in optical fibres with the transmittance 
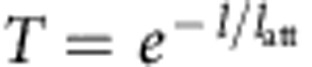
 for distance *l* (*l*_att_=22 km) from the birth towards the generation process for 
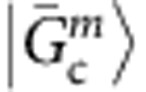
. In addition, we suppose that the optical fibres have small errors when they are used to connect distant repeater stations (*L*_0_/2 apart), and the errors of the fibre with length *L*_0_/2 can be described as an individual depolarizing channel with error probability *e*_d_. We also assume to use single-photon sources with efficiency *η*_S_, photon detectors with quantum efficiency *η*_D_ and active feedforward techniques less than 150 ns. For the choice of *L*=5,000 km (*L*=1,000 km), *L*_0_=4 km, *e*_d_=4.2 × 10^−5^, *η*_D_*η*_S_=0.95, *f*=100 kHz and *m*=24 (*m*=19), we obtain *τ*_c_=3.1 *μ*s (*τ*_c_=2.9 *μ*s), 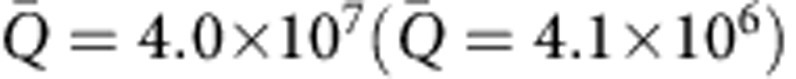, 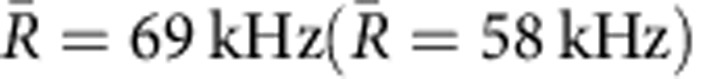 and 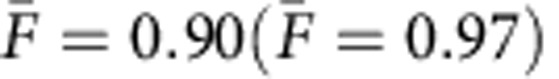 under numerical calculation to minimize 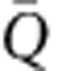 (see the detail in Supplementary Note 3). On the other hand, if Alice and Bob use the direct transmission of single photons emitted by a 10-GHz single-photon source, in order to share an entangled pair, they need to consume, on average, 5.1 × 10^98^ (5.5 × 10^19^) single photons and to take 10^81^ (175) years. This striking contrast highlights the exponential superiority of our repeater protocol to the existing photonic protocols^24^,^25^,^26^. In addition, the rate 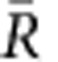 of our protocol is at least 5 order of magnitude better than those of the standard repeater schemes^6^,^7^,^8^,^11^,^14^,^15^. Moreover, our protocol is comparable to the speediest protocol of Munro *et al*.^20^ in the rate 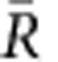, although their protocol^20^ uses not only single photons but also demanding matter qubits and their required numbers are in the same order of the consumed photons 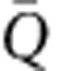 in our protocol (see Supplementary Note 3).

## Discussion

The existing quantum repeater theories require matter quantum memories to have properties such as infinite coherence time^6^,^7^,^8^, an on-demand emission of a single photon^6^,^7^,^8^,^11^,^14^, combination with a single-photon source^6^,^20^, DiVincenzo's all the criteria^30^ beyond his five criteria for universal quantum computation^4^,^9^,^11^,^12^,^13^,^14^,^15^,^16^,^17^,^18^,^19^,^20^,^21^ and combination with a reversible quantum interface^37^,^38^ between photons with different wave lengths^4^,^6^,^7^,^8^,^9^,^10^,^11^,^12^,^13^,^14^,^15^,^16^,^17^,^18^,^19^,^20^,^21^. Some of these key properties have been demonstrated via very recent experimental advances^32^ in matter quantum memories but still are challenging to be satisfied together. In contrast, our all-photonic protocol does not use matter quantum memories at all, and all the basic optical elements have already been developed in a main stream^5^ towards a quantum computer^29^ and in conjunction with an all optical approach^49^ in conventional communications. Actually, even our protocol requires that various technologies can be made to work together, for example, to generate a large-scale photonic cluster state, which is still challenging. However, it is certain that our protocol greatly reduces the number of requisites for quantum repeaters and opens up a completely new route. Even from a fundamental viewpoint, the all-photonic nature of our theory enables single photons to fully describe even quantum repeaters in addition to quantum computation^29^ and boson sampling^50^, which represents the potential of single photons as unified and fair language to compare complexities of quantum information processing protocols. In fact, this feature has led to the first rigorous proof that a quantum repeater is much simpler than a quantum computer. We have only just begun to grasp the full implications of all-photonic quantum repeaters: for example, a proposal for a good single-photon source (for example, like the one^51^), a proof-of-principle experiment with photons with a telecom wavelength, a more experimentally oriented modification of our protocol, a more robust improvement against noise such as equipping our protocol with full fault tolerance (for example, a combination with cluster-state-based entanglement purification^16^ as in Supplementary Discussion) and a generalization for a general network topology including a two-dimensional lattice or an irregular two-dimensional graph will lead to an attractive new twist.

## Author contributions

K.A. conceived the first version of the main concept of all-photonic quantum repeaters during his visit to H.-K.L.'s group at the University of Toronto with K.T. Then, all the authors contributed to the refinement and generalization of the main concept and its presentation and writing of the present paper.

## Additional information

**How to cite this article:** Azuma, K. *et al*. All-photonic quantum repeaters. *Nat. Commun.* 6:6787 doi: 10.1038/ncomms7787 (2015).

## Supplementary Material

Supplementary Information Supplementary Figures 1-5, Supplementary Notes 1-3, Supplementary Discussion and Supplementary References
