# Supplementary material for: All-photonic quantum repeaters
Source: Nat Commun. 2015 Apr 15;6:6787. doi: 10.1038/ncomms7787 (PMC4410623; doi:10.1038/ncomms7787)
Supplement: Supplementary Information — Supplementary Figures 1-5, Supplementary Notes 1-3, Supplementary Discussion and Supplementary References [file ncomms7787-s1.pdf]

Supplementary Figures

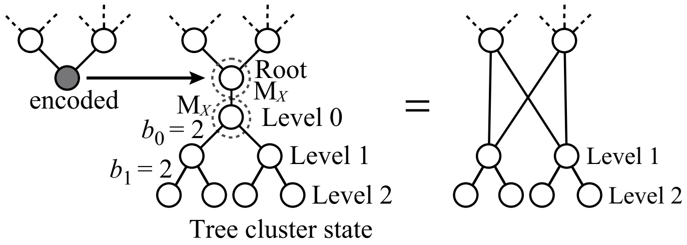

Supplementary Figure 1: Example of the encoding of a qubit on a tree cluster state with branching parameters $b_0 = b_1 = 2$. The grey qubit corresponds to the encoded qubit. X-basis measurements on the root and 0th-level qubits should be applied *in advance* to complete the encoding, linking every 1st-level qubit to all the qubits that have been connected to the encoded qubit.

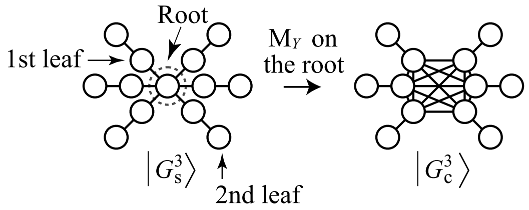

Supplementary Figure 2: Star-like cluster state $|G_s^m\rangle$ (for the case of $m = 3$). The Y-basis measurement M_Y on the root qubit transforms the state into a complete-like cluster state $|G_c^m\rangle$, up to local unitary operations [7].

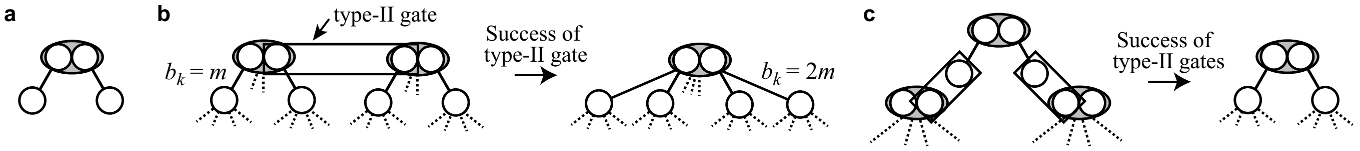

Supplementary Figure 3: Varnava *et al.*'s protocol for producing a tree cluster state [2, 3]. **a,** A 2-tree that is the simplest tree cluster state with a redundantly encoded root qubit (composed of two qubits) and branching parameter $\{2\}$. **b,** Successful application of the type-II gate to the pair of the redundantly encoded root qubits of tree cluster states with the highest branching parameter $b_k = m$ produces a one with the highest branching parameter $b_k = 2m$. **c,** Adding a higher level to tree cluster states with branching parameters $\{b_k, b_{k+1}, \ldots, b_l\}$ via the type-II gates with the help of a 2-tree. If the type-II gates succeed, we obtain $\{2, b_k, b_{k+1}, \ldots, b_l\}$.

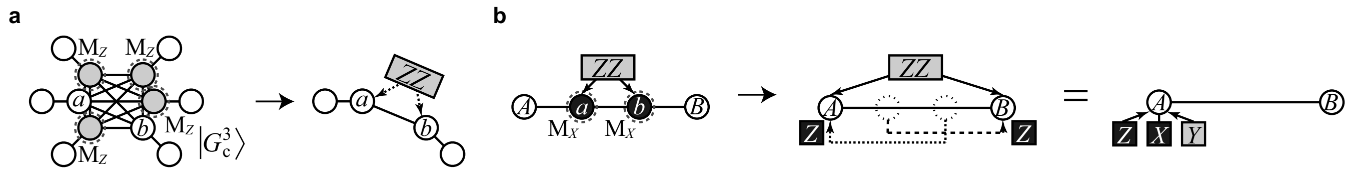

Supplementary Figure 4: Error propagations in cluster states. **a,** Error propagation of Z-basis measurements on $2(m-1)$ 1st-leaf qubits in state $|G_c^m\rangle$ ($m=3$). Depending on the total parity k of the Z-basis measurements, unitary operation $\hat{Z}_{ab}^k$ ($\hat{Z}_{ab} := \hat{Z}_a\hat{Z}_b$) is performed. The error of the Z-basis measurements thus leads to a phase-flip channel $\Lambda^{Z_{ab}}$. **b,** Error propagation of two adjacent loss-tolerant X-basis measurements on qubits ab that connect two-end qubits AB linearly. The measurement outcome k_a (k_b) on qubit a (b) is correlated with the phase flip $\hat{Z}_B^{k_a}$ ($\hat{Z}_A^{k_b}$), implying that the measurement error leads to the phase-flip channel on qubit B (A). Since the stabilizers for a bipartite cluster state are $\hat{X}_A\hat{Z}_B$ and $\hat{Z}_A\hat{X}_B$, $\hat{Z}_B$ ($\hat{Z}_{AB}$) has the same action with $\hat{X}_A$ ($\hat{Y}_A$) for the state.

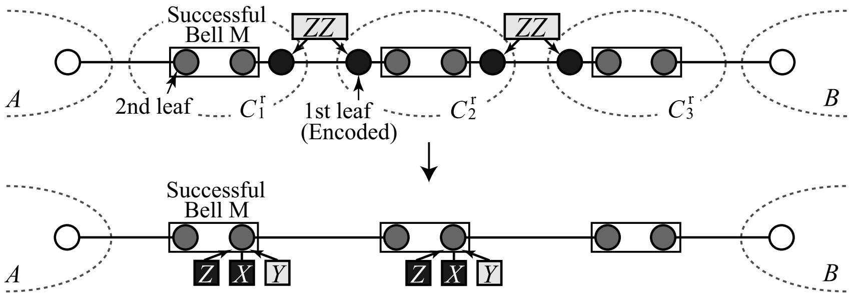

Supplementary Figure 5: Error propagation in the success case of our repeater protocol defined in Fig. 3 ($n=2$). The Bell measurement here is defined in Fig. 2**a**. The 2nd-leaf qubits are subject to the depolarization represented by $\mathcal{E}$ (without exception), and we describe this effect by colouring the 2nd-leaf qubits in grey.

Supplementary Note 1. LOSS-TOLERANT SINGLE-QUBIT MEASUREMENT

In our repeater protocol defined in Fig. 3, we use the protocol of Varnava *et al.* [1] to perform Z-basis and X-basis measurements on the 1st-leaf qubits of the encoded complete-like cluster state $|\bar{G}_{\rm c}^{m}\rangle$. This protocol has originally been proposed to execute a single-qubit measurement of an observable $\hat{A}(\alpha)=\cos\alpha\hat{X}+\sin\alpha\hat{Y}$ on a qubit in a cluster state with an arbitrary high success probability under the loss, and it *does not* have robustness against general errors in general. However, if this protocol is used only to implement a Z-basis or X-basis measurement as in our repeater protocol, the protocol can be so equipped with a majority vote as to have robustness against general errors, as briefly mentioned by Varnava *et al.* [1]. This special robustness is utilized in our repeater protocol to overcome the losses and small errors for photons in the transmission between adjacent source and receiver nodes. In this Supplementary Note, we prove the special robustness of Varnava *et al.*'s protocol [1], through reviewing basic results of Ref. [1] first, then elaborating the analysis of the effects of general errors on the protocol equipped with the majority vote and finally showing the numerical examples.

Basic results of Ref. [1]. Let us begin by briefly reviewing the basic results shown in Ref. [1]. The loss-tolerant measurement [1] is a way to execute the single-qubit measurement of an observable $\hat{A}(\alpha)=\cos\alpha\hat{X}+\sin\alpha\hat{Y}$ on a qubit in a cluster state under the loss. The qubit to be equipped with the function of the loss-tolerant measurement, called encoded qubit, should be replaced by the root qubit of a tree cluster state with branching parameters $\{b_i\}_{i=0,1,\dots,l}$ like Supplementary Fig. 1, and, further, X-basis measurements on the root and 0th-level qubits should be applied *in advance*. These X-basis measurements connect every 1st-level qubit to all the qubits that have been linked to the encoded qubit. The number $Q_{\rm L}$ of qubits in the tree cluster state is

$$Q_{\rm L}=\sum_{j=0}^{l}\prod_{i=0}^{j}b_i. \tag{1}$$

The loss-tolerant measurement is executed by applying proper single-qubit measurements on the qubits below the 1st level in the tree. According to Ref. [1], for (individual) loss probability ϵ_0, the success probability $P_{\rm L}$ of the loss-tolerant measurement of observable $\hat{A}(\alpha)$ is described by

$$P_{\rm L}=[(1-\epsilon_0+\epsilon_0R_1)^{b_0}-(\epsilon_0R_1)^{b_0}](1-\epsilon_0+\epsilon_0R_2)^{b_1}, \tag{2}$$

where R_k is the success probability of implementing an indirect Z-basis measurement on any given qubit found in the kth level of the tree, specified by

$$R_k=1-[1-(1-\epsilon_0)(1-\epsilon_0+\epsilon_0R_{k+2})^{b_{k+1}}]^{b_k} \tag{3}$$

through $1\le k\le l$, $R_{l+1}:=0$ and $b_{l+1}:=0$. Note that $P_{\rm L}$ of Eq. (2) can be determined by solving the equations of (3) recursively. The success probability $P_{\rm L}$ of this measurement can be made arbitrary close to unity as long as $\epsilon_0<0.5$, and it is numerically shown [1, 3] to be

$$Q_{\rm L}\simeq{\rm poly}\ln\frac{1}{1-P_{\rm L}}\simeq\left(\ln\frac{1}{1-P_{\rm L}}\right)^{4.5}. \tag{4}$$

Note that, for a qubit in the tree cluster state, we can perform a Z-basis measurement even if we lose the qubit. This *indirect Z-basis measurement* is essential for the protocol of Varnava *et al.* [1]. Let N_a be the set of qubits that are (directly) connected to qubit a. The indirect Z-basis measurement on a qubit A in the kth level of the tree is achieved by the joint success of the direct X-basis measurement on *any* qubit $B\in N_A$ in the $(k+1)$th level and direct or indirect Z-basis measurements on all the b_{k+1} qubits $\{C_i^B\}_{i=1,\dots,b_{k+1}}$ that are in the $(k+2)$th level and in N_B. The working principle of this scheme with respect to a qubit $B\in N_A$ in the $(k+1)$th level is based on the fact that the tree cluster state is stabilized [6] by operator $\hat{Z}_A\hat{X}_B\otimes_{i=1,\dots,b_{k+1}}\hat{Z}_{C_i^B}$ and the measurement outcome of the observable $\hat{Z}_A$ can thus be guessed by the parity of the observable $\hat{X}_B\otimes_{i=1,\dots,b_{k+1}}\hat{Z}_{C_i^B}$. Since the protocol with respect to another qubit $B'\in N_A$ in the same $(k+1)$th level works similarly and independently of that on the qubit B, the indirect Z-basis measurement on qubit A succeeds when, under the parallel implementation of the schemes with respect to all the $(k+1)$th-level qubits in N_A, at least, one of them succeeds. In addition, the independence of the schemes allows us to use a majority vote [1] to increase the fidelity of the measurement outcome of the indirect Z-basis measurement on qubit A. These properties are essential for greatly increasing the success probability of the loss-tolerant measurement as well as the robustness in the case of the measurement of observable $\hat{Z}$ or $\hat{X}$ against

depolarization for the physical qubits (as will be seen in the rest of this Supplementary Note 1).

Error analysis. In our repeater scheme, we use the protocol of Varnava *et al.* to perform a Z-basis or X-basis measurement under loss as well as general errors. Thus, assuming the existence of individual depolarization for qubits in the tree cluster state with branching parameters $\{b_i\}_{i=0,1,\ldots,l}$, we investigate the effects of the errors for the loss-tolerant Z-basis and X-basis measurements under the use of a majority vote [1].

The depolarizing channel $\mathcal{E}_A$ for qubit A in state $\hat{\rho}$ is defined by

$$\mathcal{E}_A(\hat{\rho}) = (1-e_{\rm d})\hat{\rho} + \frac{e_{\rm d}}{3}(\hat{X}_A\hat{\rho}\hat{X}_A + \hat{Y}_A\hat{\rho}\hat{Y}_A + \hat{Z}_A\hat{\rho}\hat{Z}_A), \tag{5}$$

where $e_{\rm d}$ is the error probability of this channel. If we consider a Pauli measurement on the qubit A in state $\mathcal{E}_A(\hat{\rho})$ (this is actually the case for our repeater scheme), the error probability $e_{\rm m}$ of this measurement is

$$e_{\rm m} = \frac{2}{3}e_{\rm d}. \tag{6}$$

Then, our error model considered in what follows can be specified as the one where all the qubits in the tree cluster state are subject to a depolarizing channel, independently, and the goal here is to evaluate the effects of these errors for the loss-tolerant Z-basis and X-basis measurements.

We first consider the expectation value of the error probability $e_{{\rm I}_k}$ of the indirect Z-basis measurement on a qubit A in the kth level in the tree. As noted in the previous section, this measurement outcome of observable $\hat{Z}_A$ is guessed by the parity of the observable $\hat{X}_B \otimes_{i=1,\ldots,b_{k+1}} \hat{Z}_{C_i^B}$, where B is a $(k+1)$th-level qubit in N_A and $\{C_i^B\}_{i=1,\ldots,b_{k+1}}$ are $(k+2)$th-level qubits in N_B. The success probability S_k of the protocol to find out the parity of the observable $\hat{X}_B \otimes_{i=1,\ldots,b_{k+1}} \hat{Z}_{C_i^B}$ is

$$S_k = (1-\epsilon_0)(1-\epsilon_0+\epsilon_0 R_{k+2})^{b_{k+1}}. \tag{7}$$

This event requires the joint success of direct or indirect Z-basis measurements on all the qubits $\{C_i^B\}_{i=1,\ldots,b_{k+1}}$. Suppose that, among the qubits $\{C_i^B\}_{i=1,\ldots,b_{k+1}}$, l_k qubits output only the outcomes of direct Z measurements with error probability $e_{\rm m}$, while the other $(b_{k+1}-l_k)$ qubits have the measurement outcomes of the indirect Z-basis measurements with average error probability $\bar{e}_{{\rm I}_{k+2}}$. *Here we assume that the measurement outcome of the indirect Z-basis measurement on a qubit is preferentially accepted if both of the direct and indirect measurements on it succeed.* Then, in addition to the error probability of the direct X-basis measurement on qubit B, the average error probability of guessing the parity of the observable $\hat{X}_B \otimes_{i=1,\ldots,b_{k+1}} \hat{Z}_{C_i^B}$, i.e., the average error probability $\bar{e}_{{\rm I}_k|B}$ of the indirect Z-basis measurement on qubit A from the measurements on the qubit B and on the $(k+2)$th-level qubits around B, is estimated as

$$\bar{e}_{{\rm I}_k|B} = \sum_{l_k=0}^{b_{k+1}} \begin{pmatrix} b_{k+1} \\ l_k \end{pmatrix} \left(1-\frac{R_{k+2}}{1-\epsilon_0+\epsilon_0 R_{k+2}}\right)^{l_k} \left(\frac{R_{k+2}}{1-\epsilon_0+\epsilon_0 R_{k+2}}\right)^{b_{k+1}-l_k} \frac{1-(1-2e_{\rm m})^{1+l_k}(1-2\bar{e}_{{\rm I}_{k+2}})^{b_{k+1}-l_k}}{2}. \tag{8}$$

This is the average error probability of the indirect Z-basis measurement scheme with respect to a qubit $B\in N_A$ in the $(k+1)$th level. Note that we can also obtain the outcome of observable Z_A by running such an indirect measurement scheme with respect to another qubit $B'\in N_A$ in the $(k+1)$th level. Therefore, by performing all the schemes on all the $(k+1)$th-level qubits in N_A, we can take a majority vote over all the guessing outcomes of observable Z_A that are obtained by all the successful ones. Suppose that there are $m_k(\geq 1)$ successful ones, which occurs with probability

$$T_k(m_k) := \begin{pmatrix} b_k \\ m_k \end{pmatrix} S_k^{m_k}(1-S_k)^{b_k-m_k}. \tag{9}$$

Then, the average guessing probability $\bar{e}_{{\rm I}_k|m_k}$ of the measurement outcome of observable $\hat{Z}_A$ from the majority vote is

$$\bar{e}_{{\rm I}_k|m_k} = \begin{cases} \sum_{j=\lceil m_k/2\rceil}^{m_k} \begin{pmatrix} m_k \\ j \end{pmatrix} (\bar{e}_{{\rm I}_k|B})^j(1-\bar{e}_{{\rm I}_k|B})^{m_k-j} & (m_k \text{ is odd}), \\ \sum_{j=\lceil m_k/2\rceil}^{m_k-1} \begin{pmatrix} m_k-1 \\ j \end{pmatrix} (\bar{e}_{{\rm I}_k|B})^j(1-\bar{e}_{{\rm I}_k|B})^{m_k-1-j} & (m_k \text{ is even}), \end{cases} \tag{10}$$

where $\lceil x \rceil$ is the smallest integer among integers that are greater than or equal to x. Therefore, the average error probability $\bar{e}_{\mathrm{I}_k}$ is

$$\bar{e}_{\mathrm{I}_k} = \frac{1}{R_k} \sum_{m_k=1}^{b_k} T_k(m_k) \bar{e}_{\mathrm{I}_{k|m_k}}, \tag{11}$$

where we used

$$R_k = \sum_{m_k=1}^{b_k} T_k(m_k). \tag{12}$$

$\{\bar{e}_{\mathrm{I}_k}\}_{k=0,1,\ldots,l}$ can be derived by solving Eqs. (8)-(11) recursively.

Let us move to the error analysis for the loss-tolerant Z-basis measurement. This measurement succeeds when direct or indirect Z-basis measurements on all the 1st-level qubits succeed (c.f., Supplementary Fig. 1). Thus, the success probability P_Z of the loss-tolerant Z-basis measurement is described by

$$P_Z = (1 - \epsilon_0 + \epsilon_0 R_1)^{b_0}. \tag{13}$$

On the other hand, since all the 1st-level qubits are linked to all the qubits that have been connected to the encoded qubit as in Supplementary Fig. 1, the average error probability $\bar{e}_Z$ of the loss-tolerant Z-basis measurement is

$$\bar{e}_Z = \sum_{l=0}^{b_0} \begin{pmatrix} b_0 \\ l \end{pmatrix} \left(1 - \frac{R_1}{1 - \epsilon_0 + \epsilon_0 R_1}\right)^l \left(\frac{R_1}{1 - \epsilon_0 + \epsilon_0 R_1}\right)^{b_0 - l} \frac{1 - (1 - 2e_{\mathrm{m}})^l (1 - 2\bar{e}_{\mathrm{I}_1})^{b_0 - l}}{2}. \tag{14}$$

Let us proceed to the error analysis for the loss-tolerant X-basis measurement. This measurement succeeds if the direct X-basis measurement on any 1st-level qubit B_1 and direct or indirect Z-basis measurements on all the 2nd-level qubits $\{C_i^{B_1}\}_{i=1,\ldots,b_1} =: C(B_1)$ in N_{B_1} succeed, i.e., if we know the parity of $\hat{X}_{B_1} \hat{Z}_{C(B_1)}$, where $\hat{Z}_N := \otimes_{i \in N} \hat{Z}_i$. To see this, let $\{B_i\}_{i=1,\ldots,b_0} =: B$ be 1st-level qubits, let $\{A_i\}_i =: A$ be qubits that have been connected to the encoded qubit, i.e., $A = N_{B_i} \setminus C(B_i)$ (c.f., Supplementary Fig. 1). Through the measurement of parity $k_1 (= 0, 1)$ of observable $\hat{X}_{B_1} \hat{Z}_{C(B_1)}$, the stabilizers for the initial cluster state are renewed as

$$\begin{cases} \hat{X}_{A_i} \hat{Z}_B \hat{Z}_{N_{A_i} \setminus B}, \\ \hat{Z}_A \hat{X}_{B_i} \hat{Z}_{C(B_i)}, \end{cases} \to \begin{cases} \hat{X}_{A_1} \hat{X}_{A_i} \hat{Z}_{N_{A_1} \setminus B} \hat{Z}_{N_{A_i} \setminus B} \ \ (i \neq 1), \\ (-1)^{k_1} \hat{Z}_A, \\ (-1)^{k_1} \hat{X}_{B_1} \hat{Z}_{C(B_1)}, \\ (-1)^{k_1} \hat{X}_{B_i} \hat{Z}_{C(B_i)} \ \ (i \neq 1). \end{cases} \tag{15}$$

The stabilizers in the first and second rows of the right-hand side (RHS) of this equation correspond to the desired backaction that is the same as that of the direct X-basis measurement on the encoded qubit. The stabilizers in the last row of the RHS indicate that $\{B_i\}_{i=2,\ldots,b_0}$ are decoupled from qubits in A and the parity of observable $\hat{X}_{B_i} \hat{Z}_{C(B_i)}$ $(i \neq 1)$ is the same as k_1, These consequences suggest that the loss-tolerant X-basis measurement succeeds if we succeed in finding out one of the parities of $\{\hat{X}_{B_i} \hat{Z}_{C(B_i)}\}_{i=1,\ldots,b_0}$, and that we can use a majority vote like the indirect Z-basis measurement on a qubit in a level. Therefore, the success probability P_X of the loss-tolerant X-basis measurement is

$$P_X = R_0, \tag{16}$$

and the average error probability is

$$\bar{e}_X = \bar{e}_{\mathrm{I}_0}. \tag{17}$$

Note that the success probability of the loss-tolerant measurement for general observable $\hat{A}(\alpha)$ is less than those for observables $\hat{Z}$ and $\hat{X}$, i.e., $P_{\mathrm{L}} \leq P_Z$ and $P_{\mathrm{L}} \leq P_X$ [c.f., Eqs. (2), (13) and (16)]. In addition, the average error probability $\bar{e}_{\mathrm{L}}$ of the loss-tolerant measurement for general observable $\hat{A}(\alpha)$ is in the order of e_{m} owing to the direct contribution of the error of the direct measurement of observable $\hat{A}(\alpha)$ on a 1st-level qubit [1]. This is in contrast to the error probabilities $\bar{e}_Z$ and $\bar{e}_X$ that have a mechanism—a majority vote—to greatly suppress the error induced by individual depolarization. *These contrasts in error probability and success probability between the protocol for observable $\hat{Z}$ or $\hat{X}$ and that for general observable $\hat{A}(\alpha)$ represent a notable difference between quantum repeaters and*

quantum computing, in the sense that quantum repeaters are possible without such a general single-qubit measurement like our protocol but quantum computation is not [5].

Numerical examples. To ensure the special robustness against general errors that appears in the cases of Z-basis and X-basis measurements, here we present numerical examples of P_Z, P_X, $P_{\rm L}$, $\bar{e}_Z$ and $\bar{e}_X$. (I) For $\epsilon_0 \simeq 0.20$ and $e_{\rm m} = 2e_{\rm d}/3 = 2.8 \times 10^{-5}$, the encoded qubit with branching parameters $\{b_0, b_1, b_2\} = \{16, 14, 1\}$ ($\{b_0, b_1, b_2\} = \{11, 11, 1\}$), which is composed of $Q_{\rm L} = 464$ ($Q_{\rm L} = 253$) qubits, gives $1 - P_Z = 1.8 \times 10^{-6}$ ($1 - P_Z = 2.5 \times 10^{-5}$), $1 - P_X = 5.9 \times 10^{-5}$ ($1 - P_X = 3.2 \times 10^{-4}$), $1 - P_{\rm L} = 0.43$ ($1 - P_{\rm L} = 0.35$), $\bar{e}_Z = 1.6 \times 10^{-7}$ ($\bar{e}_Z = 1.4 \times 10^{-6}$) and $\bar{e}_X = 2.5 \times 10^{-6}$ ($\bar{e}_X = 8.3 \times 10^{-6}$). (II) For $\epsilon_0 \simeq 0.27$ and $e_{\rm m} = 2e_{\rm d}/3 = 5.6 \times 10^{-5}$, the encoded qubit with branching parameters $\{b_0, b_1, b_2\} = \{17, 28, 2\}$ ($\{b_0, b_1, b_2\} = \{12, 23, 2\}$), which is composed of $Q_{\rm L} = 1445$ ($Q_{\rm L} = 840$) qubits, gives $1 - P_Z = 4.9 \times 10^{-6}$ ($1 - P_Z = 3.7 \times 10^{-5}$), $1 - P_X = 1.1 \times 10^{-4}$ ($1 - P_X = 5.6 \times 10^{-4}$), $1 - P_{\rm L} = 0.43$ ($1 - P_{\rm L} = 0.37$), $\bar{e}_Z = 5.2 \times 10^{-7}$ ($\bar{e}_Z = 2.8 \times 10^{-6}$) and $\bar{e}_X = 1.5 \times 10^{-5}$ ($\bar{e}_X = 4.7 \times 10^{-5}$). These data sets (I) and (II) are obtained by numerical calculation based on Eqs. (1)-(3), (13), (14), (16) and (17), and they will be used in Supplementary Note 3 to estimate the performance of our repeater protocol for $L_0 = 4$ km and $L_0 = 8$ km numerically. For those choices of the parameters, the success probabilities P_Z and P_X for Z-basis and X-basis measurements are *much higher* than the success probability $P_{\rm L}$ for general observable $\hat{A}(\alpha)$. More specifically, the branching parameters chosen here are too small to obtain $P_{\rm L} > 1 - \epsilon_0$, but they are large enough to achieve our quantum repeater protocol that uses only the loss-tolerant Z-basis and X-basis measurements. In addition, the *smaller* values of $\bar{e}_Z$ and $\bar{e}_X$ than $e_{\rm m}$ imply that the loss-tolerant Z-basis or X-basis measurement is robust even against individual errors on physical qubits, which is in contrast to the general observable $\hat{A}(\alpha)$. We also note that the greater the branching ratios the trees have, the lower the failure probability and error probability of the loss-tolerant Z-basis or X-basis measurement [1] become.

Supplementary Note 2. PREPARATION OF AN ENCODED COMPLETE-LIKE CLUSTER STATE $|\bar{G}_{\rm c}^m\rangle$

In this Supplementary Note, we show that the state $|\bar{G}_{\rm c}^m\rangle$ can be prepared in *very-short constant* time $\tau_{\rm c}$ irrespective of the existence of photon loss, via a *synchronized parallel* procedure of Varnava *et al.* [2, 3] based on linear optical elements, single-photon sources, photon detectors, optical switches and a high-speed active feedforward technique alone. The synchronization is applied to the generation of single photons, the application of the single-qubit and two-qubit measurements (based on linear optical elements and photon detectors) and adaptive routing of single photons that succeeded as candidates for the final state $|\bar{G}_{\rm c}^m\rangle$. The parallelism based on this synchronization makes all the probabilistic steps provide enough candidates to finally present at least one successful state $|\bar{G}_{\rm c}^m\rangle$ after time $\tau_{\rm c}$. The preparation time $\tau_{\rm c}$ estimated here will be translated into the corresponding inherent loss probability for individuals of photons in state $|\bar{G}_{\rm c}^m\rangle$, which will be used to estimate the performance of our repeater protocol in Supplementary Note 3. Here we begin by considering the preparation of an encoded *star-like* cluster state $|\bar{G}_{\rm s}^m\rangle$ that can be transformed into the state $|\bar{G}_{\rm c}^m\rangle$.

The star-like cluster state denoted by $|G_{\rm s}^m\rangle$ is described by the graph of Supplementary Fig. 2. This state centres a single root qubit that has $2m$ arms ($m \geq 1$) composed of 1st-leaf and 2nd-leaf qubits. The state has been used as the basic unit to produce 2-dimensional or 3-dimensional cluster states [8, 9]. The star-like cluster state with 1st-leaf qubits being encoded is denoted by $|\bar{G}_{\rm s}^m\rangle$, and the encoding allows us to execute the loss-tolerant measurements on the 1st-leaf qubits. In particular, the state $|\bar{G}_{\rm s}^m\rangle$ is obtained by replacing the 1st-leaf qubits in the state $|G_{\rm s}^m\rangle$ with the root qubits of the $Q_{\rm L}$-qubit tree cluster states with branching parameters $\{b_i\}_{i=0,1,\ldots,l}$, where the root and 0th-level qubits of the $Q_{\rm L}$-qubit tree cluster states are to be subjected to X-basis measurements in order to complete the loss-tolerant encoding. We note that the whole state $|\bar{G}_{\rm s}^m\rangle$ is (not equivalent to but) similar to a tree cluster state with branching parameters $\{2m, 2, b_0, \ldots, b_l\}$. In general, such a tree cluster state can be prepared efficiently via the protocol [2, 3] of Varnava *et al.*

The protocol of Varnava *et al.* [2, 3] uses linear optical elements, single-photon sources, photon detectors, optical switches and a high-speed active feedforward technique. Suppose that the single-photon sources have efficiency $\eta_{\rm S}$ and the single-photon detectors have quantum efficiency $\eta_{\rm D}$. The high-speed active feedforward technique is used to signal the linear optical circuit to perform proper single-qubit measurements on specified single photons and to apply two-qubit measurements to specified pairs of single photons in conjunction with optical switches, dependently on the previous measurement outcomes. Suppose that $\tau_{\rm a}$ represents the time to execute a single-qubit or two-qubit measurement and the associated classical feedforward. In the estimations of the performance of our quantum repeater scheme in Supplementary Note 3, from an experimental result [10] on an active feedforward technique for the measurement-based quantum computation, $\tau_{\rm a}$ will be assumed to be 150 ns, during which single photons will be assumed to run in optical fibres with lengths of about 30 m.

To generate the star-like cluster state $|\bar{G}_{\rm s}^m\rangle$, the protocol [2, 3] proceeds as follows: (a) We first run protocols (in Fig. 1 of Supplemental Material of Ref. [2]) in parallel, each of which converts six single photons to a three-qubit GHZ state with an effective individual loss probability $1-\eta_{\rm S}/(2-\eta_{\rm D}\eta_{\rm S})$ with success probability $\eta_{\rm D}^3\eta_{\rm S}^3(2-\eta_{\rm D}\eta_{\rm S})^3/32$ [2]. (b) Then, we apply 'type-II gates'—probabilistic (destructive) Bell measurements—in parallel to all the pairs of the three-qubit GHZ states in order to obtain '2-trees'—which are the simplest tree cluster states that have a redundantly encoded root qubit (composed of two qubits) and branching parameter $\{2\}$ (c.f., Supplementary Fig. 3**a**)—in the success cases. (c) We perform the type-II gates in parallel on all the pairs of the redundantly encoded root qubits of 2^j-trees with branching parameter $\{2^j\}$ $(j=1,2,\ldots,\lceil\log_2 b_l\rceil-1)$ in order to obtain 2^{j+1}-trees with the redundantly encoded [2, 3] root qubit in the success cases (Supplementary Fig. 3**b**), which is continued until obtaining $2^{\lceil\log_2 b_l\rceil}$-trees that provide b_l-trees by cutting out surplus branches via Z-basis measurements if necessary. In general, this process has the role to increase the branching number of the highest level of tree cluster states with the redundantly encoded root qubit (c.f., Supplementary Fig. 3**b**). (d) Then, we run protocols of Supplementary Fig. 3**c** in parallel, each of which transforms a pair of b_l-trees to a tree with the redundantly encoded root qubit and branching parameters $\{2,b_l\}$ in conjunction with a 2-tree. In general, this process adds a higher level to tree cluster states (c.f., Supplementary Fig. 3**c**). (e) By running the process of Supplementary Fig. 3**b** similarly to step (c) and the process of Supplementary Fig. 3**c** similarly to step (d) alternately, we can finally obtain any tree cluster state, and thus the star-like cluster state $|\bar{G}_{\rm s}^m\rangle$ like a tree cluster state with branching parameters $\{2m,2,b_0,\ldots,b_l\}$.

As implied by the steps (a)-(e), with synchronization for the single-photon generation and adaptive routing of single photons in the linear optical circuit, Varnava *et al.*'s protocol [2, 3] proceeds like a knockout tournament that fairly deals with all the candidates for the final state $|\bar{G}_{\rm s}^m\rangle$. Thanks to this feature, Varnava *et al.*'s protocol allows us to generate the star-like cluster state $|\bar{G}_{\rm s}^m\rangle$ very fast and to assume that all the photons for $|\bar{G}_{\rm s}^m\rangle$ are subject to *individual* photon loss [2, 3].

According to the result of Refs. [2, 3], the expected number $\bar{Q}_{\rm s}$ of total single photons required to produce the state $|\bar{G}_{\rm s}^m\rangle$ (whose root qubit is actually the redundantly encoded qubit composed of two bare qubits) is bounded by

$$\bar{Q}_{\rm s}\le\frac{2\times6\times32}{\eta_{\rm S}^3\eta_{\rm D}^3(2-\eta_{\rm S}\eta_{\rm D})^3}\frac{1}{P_{\rm II}^{2l+4}}{\rm poly}(2m)\prod_{i=0}^{l}{\rm poly}(b_i),\tag{18}$$

where $P_{\rm II}$ is the success probability of the type-II fusion. On the other hand, the time $\tau_{\rm s}$ needed to prepare the state $|\bar{G}_{\rm s}^m\rangle$ is [2, 3] described by

$$\tau_{\rm s}\simeq\left(\log_2 2m+\sum_{i=0}^{l}\log_2 b_i+l+2+2\right)\tau_{\rm a}=\left(\log_2 2m+\sum_{i=0}^{l}\log_2 b_i+l+4\right)\tau_{\rm a},\tag{19}$$

where the last term $2\tau_{\rm a}$ in the first equation comes from steps (a) and (b) and the other terms in the same equation result from the other steps. The time $\tau_{\rm s}$ is just a constant that is logarithmically dependent on the branching parameters of $|\bar{G}_{\rm s}^m\rangle$ alone. The expression of $P_{\rm II}$ in Eq. (18) actually depends on the timing of the application of the corresponding type-II fusion gate, but it is at least bounded as

$$\frac{\eta_{\rm S}^2\eta_{\rm D}^2P_{\tau_{\rm a}}^{2\tau_{\rm s}/\tau_{\rm a}}}{2(2-\eta_{\rm S}\eta_{\rm D})^2}\le P_{\rm II}\le\frac{\eta_{\rm S}^2\eta_{\rm D}^2}{2(2-\eta_{\rm S}\eta_{\rm D})^2},\tag{20}$$

where $P_{\tau_{\rm a}}$ is the survival probability of a photon for the time $\tau_{\rm a}$. Note that the bound of Eq. (18) scales polynomially with the total photon number of the state $|\bar{G}_{\rm s}^m\rangle$, i.e., $2m(Q_{\rm L}+3)+2$. Through the preparation (a)-(e), the effective loss probability of the individual photons in the state $|\bar{G}_{\rm s}^m\rangle$ is $1-P_{\tau_{\rm a}}^{\tau_{\rm s}/\tau_{\rm a}}\eta_{\rm S}/(2-\eta_{\rm D}\eta_{\rm S})$.

As shown in Supplementary Fig. 2, the encoded complete-like cluster state $|\bar{G}_{\rm c}^m\rangle$ can be obtained by performing the (effective) Y-basis measurement on the (redundantly-encoded) root qubit of the state $|\bar{G}_{\rm s}^m\rangle$ as well as proper local unitary operations [note that the Y-basis measurement corresponds to a Bell measurement (on a two-dimensional subspace of the physical qubits composing the root qubit)]. We also need to perform X-basis measurements on the 1st-leaf qubits (corresponding to the root qubits of the $Q_{\rm L}$-qubit tree cluster states) in the state $|\bar{G}_{\rm s}^m\rangle$ as well as on the associated 0-level qubits in the tree, in order to complete the encoding of Supplementary Fig. 1. These measurements multiply an additional factor $[(2-\eta_{\rm S}\eta_{\rm D})/(\eta_{\rm S}\eta_{\rm D}P_{\tau_{\rm a}}^{\tau_{\rm s}/\tau_{\rm a}})]^2\times[(2-\eta_{\rm S}\eta_{\rm D})/(\eta_{\rm S}\eta_{\rm D}P_{\tau_{\rm a}}^{\tau_{\rm s}/\tau_{\rm a}})]^{4m}$ to $\bar{Q}_{\rm s}$, where the first term and the second term come from the Y-basis measurement and from the X-basis measurements, respectively. Therefore, the expected number $\bar{Q}_{\rm c}$ of total single photons needed to produce the state $|\bar{G}_{\rm c}^m\rangle$ and the time $\tau_{\rm c}$ are

$$\bar{Q}_{\rm c}=\left[\frac{2-\eta_{\rm S}\eta_{\rm D}}{\eta_{\rm S}\eta_{\rm D}P_{\tau_{\rm a}}^{\tau_{\rm s}/\tau_{\rm a}}}\right]^{4m+2}\bar{Q}_{\rm s},\tag{21}$$

$$\tau_{\rm c}=\tau_{\rm s}+\tau_{\rm a}.\tag{22}$$

In our repeater protocol of Fig. 3, the preparation of the state $|\bar{G}_{\rm c}^m\rangle$ is executed locally, and the total photon number of the state $|\bar{G}_{\rm c}^m\rangle$ is effectively determined by the loss probability in the transmission of photons between adjacent source and receiver nodes. Therefore, the photon number required to produce the state $|\bar{G}_{\rm c}^m\rangle$ for our protocol is dependent only on distances between the adjacent nodes, and is independent of the overall distance between Alice and Bob.

Supplementary Note 3. SCALING AND PERFORMANCE OF OUR QUANTUM REPEATER PROTOCOL

Here we present the detailed analysis of the performance of our all-photonic quantum repeater protocol defined in Fig. 3, using the results in Supplementary Note 1 and Supplementary Note 2. Let ϵ_0 be a probability with which the individual photon loss occurs in the local preparation of the state $|\bar{G}_{\rm c}^m\rangle$ or in the transmission of photons between adjacent source and receiver nodes, which is selected to satisfy $\epsilon_0 < 0.5$. Note that this ϵ_0 includes the loss probability during the preparation time $\tau_{\rm c}$ for $|\bar{G}_{\rm c}^m\rangle$. We also assume that every photon is subject to individual depolarization in the transmission from a sender node to the adjacent receiver node. Let $e_{\rm d}$ be the error probability caused by this depolarization [c.f., Eq. (5) and the relation with $e_{\rm m}$].

First, we present the average total photon number $\bar{Q}$ consumed in our protocol to produce an entangled pair between Alice and Bob, the scaling of $\bar{Q}$ and the average rate $\bar{R}$ of our protocol. In addition, we show a flexibility of our protocol through considering the memory time that is needed in the case of the extreme application, i.e., quantum teleportation [11]. Next, we analyse the average errors for the obtained entangled pair and give the behaviour. Finally, we present numerical examples of these quantities as well as a comparison with the speediest protocol given by Munro *et al.* [4].

Total photon number and entanglement generation rate of our repeater protocol. Here we first present the performance of our repeater protocol that is characterized by the success probability, the average rate $\bar{R}$ for producing an entangled pair between Alice and Bob and the average total photon number $\bar{Q}$ consumed in the protocol to produce the pair. Next, we show a flexibility of our protocol via considering the requirements for our repeater protocol that emerge when it is used to accomplish quantum teleportation [11]. Finally, we derive the scaling of $\bar{Q}$.

Let us begin by considering the performance of our repeater protocol. Suppose that our protocol defined in Fig. 3 succeeds. In this case, single photons in a linear cluster state connecting Alice's qubit A and Bob's qubit B are presented, which then receive X-basis measurements at all the receiver nodes. Thus, according to the rule of Fig. 2**c**, Alice and Bob's qubits AB are entangled. Since the success events occur when all the receiver nodes have at least one successful Bell measurement and all the loss-tolerant measurements on the 1st-leaf qubits succeed, the total success probability P is described by

$$P = P_Z^{2(m-1)n} P_X^{2n} [1 - (1 - P_{\rm B})^m]^{n+1}, \tag{23}$$

where P_Z is the success probability of the loss-tolerant Z-basis measurement [c.f., Eq. (13)], P_X is the success probability of the loss-tolerant X-basis measurement [c.f., Eq. (16)] and $P_{\rm B}$ is the success probability of the Bell measurement of Fig. 2**a** represented by

$$P_{\rm B} = \frac{(1-\epsilon_0)^2}{2}. \tag{24}$$

The factor 1/2 in this expression for $P_{\rm B}$ stems from the bunching effect of the photons in the simple linear-optics-based Bell measurement. By using the success probability P, the average rate $\bar{R}$ to produce an entangled pair between Alice and Bob is simply described as

$$\bar{R} = Pf, \tag{25}$$

where f is the repetition rate of the slowest device among single-photon sources, photon detectors and active feedforward techniques. Note that we can increase this rate as high as we want just by running single-photon sources as fast as required *rather than by increasing the number of the devices and using them in parallel.* This is in striking contrast to the standard quantum repeater protocols [12–23, 25–31] as in Fig. 1, where we cannot adopt such a machine-gun-like style because the protocols, at least, need to receive the heralding signals for entanglement swapping from *distant* nodes. On the other hand, the average of the total number Q of single photons consumed in our repeater protocol to produce the entangled pair is

$$\bar{Q} = \frac{2mn(Q_{\rm L}+1)+2m}{P}. \tag{26}$$

In general, *it would be better to choose all the parameters so as to minimize $\bar{Q}$.*

Note that the maximum time $t_{\max}$ throughout which photons in our protocol are expected to survive is

$$t_{\max} = \tau_{\rm c} + \frac{L_0}{2c} + 2\tau_{\rm a}, \tag{27}$$

where $\tau_{\rm c}$ is the preparation time of the state $|\bar{G}_{\rm c}^m\rangle$, $L_0/(2c)$ is the transmission time of photons from a source node to the adjacent receiver node and $2\tau_{\rm a}$ is the time needed to perform the Bell measurement and the loss-tolerant measurements in steps (ii) and (iii) of our protocol in Fig. 3. Combined with the results in Supplementary Note 2 and with a fact that every photon is finally fed into photon detectors with quantum efficiency $\eta_{\rm D}$, the effective total loss probability ϵ_0 is approximately described by

$$\epsilon_0 \simeq 1 - e^{-L_0/(2l_{\rm att})} P_{\tau_{\rm a}}^{(\tau_{\rm c}+2\tau_{\rm a})/\tau_{\rm a}} \frac{\eta_{\rm D}\eta_{\rm S}}{2-\eta_{\rm D}\eta_{\rm S}}, \tag{28}$$

where $l_{\rm att}$ is the attenuation length of the optical channel between a source node and the adjacent receiver node.

Next, let us see a flexibility of our protocol through considering the requirements for our protocol in the case of the application to quantum teleportation [11]. If we use our protocol for quantum key distribution (QKD), the protocol does not require any quantum memory as noted in the main body of our paper. However, when we use our protocol to achieve the most general quantum communication based on quantum teleportation [11], at least, the receiver, Bob, should have quantum memories, because quantum teleportation protocol itself requires [11] the sender (Alice) to send the outcome of her Bell measurement to the receiver (Bob). But, depending on the coherence time of the quantum memories, they have two choices that make a difference in the required memory time $t_{\rm mem}$ for achieving the quantum teleportation: (a) To keep the memory time $t_{\rm mem}$ to the minimum, i.e., the classical communication time L/c required in the quantum teleportation, they can set $P \simeq 1$ through the consumption of much more single photons (i.e., by increasing $\bar{Q}$), which leads to

$$t_{\rm mem} = \frac{L_0}{2c} + \tau_{\rm a} + \max\{\tau_{\rm a}, L_0/(2c)\} + \frac{L}{c} \tag{29}$$

$$\simeq \frac{L_0}{c} + \tau_{\rm a} + \frac{L}{c}, \tag{30}$$

where the first term $L_0/(2c)$ is the time needed for the transmission of photons between adjacent source and receiver nodes, $\tau_{\rm a}$ in the second term [in the third term $(\max\{\tau_{\rm a}, L_0/(2c)\})$] is the time for performing the Bell measurements in step (ii) [the loss-tolerant measurements in step (iii)] of our protocol of Fig. 3, $L_0/(2c)$ in the third term is the transmission time of the heralding signal from Alice's adjacent receiver node to Alice and the final term L/c is the time needed by quantum teleportation protocol. Since $\tau_{\rm a} \leq L_0/(2c)$ in practice, the approximation (30) holds. On the other hand, (b) they can start quantum teleportation, upon receiving the signals for heralding the success of the repeater protocol from receiver nodes, making the required memory time $t_{\rm mem}$ be

$$t_{\rm mem} = \frac{L_0}{2c} + 2\tau_{\rm a} + \frac{L - L_0/2}{c} + \frac{L}{c} = 2\tau_{\rm a} + \frac{2L}{c}, \tag{31}$$

where the term $(L - L_0/2)/c$ is the classical communication time between Alice and the receiver node adjacent to Bob for confirming the success of the repeater protocol. The strategy (a) is advantageous when the coherence time of the quantum memories is short, while the strategy (b) has a merit that the total number $\bar{Q}$ of single photons consumed in our repeater protocol can be set to the minimum. However, in most of the cases, it would be better to adopt the strategy (b) from the following reason: If Alice and Bob try to accomplish quantum teleportation, Bob must have quantum memories with coherence time longer than classical communication time between them (that is required in the quantum teleportation protocol) at least, L/c, as in Eq. (30). Thus, in such an era when Alice and Bob want to achieve quantum teleportation, the additional coherence time $(L - L_0/2)/c$ in Eq. (31) must not be the problem for them, implying that it would be better to adopt the strategy (b).

We stress that, if we use a conventional quantum repeater protocol [12–33] based on quantum memories as described in Fig. 1, the required memory time $t_{\rm mem}$ includes, at least, further additional factors that come from the classical communication time to trigger the next round of entanglement swapping as shown in Fig. 1, irrespectively of the application (QKD or quantum teleportation). In addition, in the standard quantum repeater protocols for $L =$ 1000 km, $t_{\rm mem}$ has been estimated [13, 15, 20, 27], at least, 1 s. This is in a striking contrast to our protocol that suppresses the memory time $t_{\rm mem}$, which required only in the case of quantum teleportation, to about 10 ms even in the strategy (b) for $L = 1000$ km. These facts suggest an advantage to use our protocol instead of the conventional quantum repeaters even if we enter an era when we have a matter quantum memory, in the sense that the smaller $t_{\rm mem}$ of our protocol should lead to smaller errors (depolarization or dephasing) of the quantum memories. Moreover,

$t_{\rm mem}$ of our protocol with the strategy (a) becomes the same as the speediest repeater protocol [4] based on matter qubits at repeater nodes. Therefore, our protocol is flexible, and it has a distinguished advantage over the standard quantum repeater protocols with respect to the required memory time $t_{\rm mem}$.

We further note that the flexibility seen in the strategies (a) and (b) extends the range of the applications of our all photonic quantum repeaters. For example, even if we want to use our protocol as an almost deterministic entanglement supplier for accomplishing another quantum information processing protocols, such as nonlocal measurements [34, 35] and cheating strategies [36, 37] in position-based quantum cryptography [38], we can achieve this *in a memory-less fashion*, at least, by adopting strategy (a). Thus, the flexibility makes any kind of quantum communication scheme possible with our all-photonic quantum repeater system alone.

Finally, let us derive the scaling of $\bar{Q}$ for the distance L between Alice and Bob. Here, instead of $\bar{Q}$, for simplicity, we consider the scaling of an upper bound from $P_X \geq P_{\rm L}$ and $P_Z \geq P_{\rm L}$,

$$\bar{Q}_{\rm upp} := \frac{2mn(Q_{\rm L}+1)+2m}{P_{\rm L}^{2mn}[1-(1-P_{\rm B})^m]^{n+1}} (\geq \bar{Q}), \tag{32}$$

for a specific choice of the parameters,

$$m \simeq \log_{1-P_{\rm B}}\left[\frac{2n\ln P_{\rm L}}{2n\ln P_{\rm L}+(n+1)\ln(1-P_{\rm B})}\right], \tag{33}$$

$$P_{\rm L} = (1-P_{\rm B})^{\frac{x}{n}}, \tag{34}$$

where x is a positive constant. Note that we can choose any $x>0$ as long as $\epsilon_0 < 0.5$. Then, combined with Eq. (4), the upper bound $\bar{Q}_{\rm upp}$ becomes

$$\bar{Q}_{\rm upp} \simeq 2\left[n\left(\left[\ln\frac{1}{1-(1-P_{\rm B})^{\frac{x}{n}}}\right]^{4.5}+1\right)+1\right]\left(1+\frac{n+1}{2x}\right)^{2x}\left(1+\frac{2x}{n+1}\right)^{n+1}\log_{1-P_{\rm B}}\left(\frac{2x}{2x+(n+1)}\right). \tag{35}$$

Since $n = L/L_0 - 1$, it is concluded that the average number $\bar{Q}$ of the total single photons required to produce an entangled pair scales only polynomially with distance L.

Error analysis for our repeater protocol. Here we consider the error probabilities for the obtained entangled pair AB in the success case of our repeater protocol. We further derive the behaviour of these error probabilities.

In the success case of our repeater protocol, $2(m-1)$ 1st-leaf qubits in the encoded complete-like cluster state $|\bar{G}_{\rm c}^m\rangle$ receive the loss-tolerant Z-basis measurements with average error probability $\bar{e}_Z$. We start by considering the effect of the error that is caused by this measurement process. Suppose that we perform a Z-basis measurement on a qubit A in a cluster state. Then, note that the cluster state is stabilized by operators $\hat{X}_A\hat{Z}_{N_A}$ and $\hat{X}_i\hat{Z}_A\hat{Z}_{N_i\setminus A}$ for $i \in N_A$ [6], where $\hat{Z}_N := \otimes_{i\in N}\hat{Z}_i$ for a set N of qubits. Through the Z-basis measurement on qubit A, depending on its outcome $k(=0,1)$, these stabilizers are renewed as $(-1)^k\hat{Z}_A$ and $(-1)^k\hat{X}_i\hat{Z}_{N_i\setminus A}$ for $i\in N_A$, which stabilize the cluster state after the measurement. This fact implies that the measurement outcome k is correlated with the application of unitary $\hat{Z}_{N_A}^k$. Thus, non-zero error probability e_Z of the Z-basis measurement on qubit A leads to a channel $\Lambda_{1-2e_Z}^{Z_{N_A}}$, where

$$\Lambda_{1-2e}^{W_C}(\hat{\rho}) := (1-e)\hat{\rho} + e\hat{W}_C\hat{\rho}\hat{W}_C = \frac{1+(1-2e)}{2}\hat{\rho} + \frac{1-(1-2e)}{2}\hat{W}_C\hat{\rho}\hat{W}_C \tag{36}$$

for a Pauli operator $\hat{W}_C$ on system C. As a result, as for the loss-tolerant Z-basis measurements on $2(m-1)$ 1st-leaf qubits in state $|\bar{G}_{\rm c}^m\rangle$, the effect of the measurement error $\bar{e}_Z$ is described by phase-flip channel $\Lambda_{(1-2\bar{e}_Z)^{2m-2}}^{Z_{ab}}$ on average, where a and b are the remaining 1st-leaf two qubits. This result is summarized as in Supplementary Fig. 4**a**.

Next, we consider error propagation caused by two adjacent loss-tolerant X-basis measurements with error probability $\bar{e}_X$ on the remaining 1st-leaf qubits ab (c.f., Supplementary Fig. 4**b**). Here let us call the 2nd-leaf qubits A and B as defined in Supplementary Fig. 4**b**. We start by noting that the initial state is stabilized by operators $\hat{X}_A\hat{Z}_a$, $\hat{Z}_A\hat{X}_a\hat{Z}_b$, $\hat{Z}_a\hat{X}_b\hat{Z}_B$ and $\hat{Z}_b\hat{X}_B$. When we assume to obtain outcomes $k_a(=0,1)$ and $k_b(=0,1)$ for the respective ideal X-basis measurements on qubits a and b, these stabilizers are converted to $(-1)^{k_a}\hat{X}_a$, $(-1)^{k_b}\hat{X}_b$, $(-1)^{k_b}\hat{X}_A\hat{Z}_B$ and $(-1)^{k_a}\hat{Z}_A\hat{X}_B$. This implies that the measurement outcome k_a (k_b) is correlated with the application of unitary $\hat{Z}_B^{k_a}$ ($\hat{Z}_A^{k_b}$). Thus, the non-zero average error probability $\bar{e}_X$ of the loss-tolerant X-basis measurement on qubit a (b) leads to a phase-flip channel $\Lambda_{1-2\bar{e}_X}^{Z_B}$ ($\Lambda_{1-2\bar{e}_X}^{Z_A}$), and the inherent phase-flip channel $\Lambda_{(1-2\bar{e}_Z)^{2m-2}}^{Z_{ab}}$ on qubits ab becomes

phase-flip channel $\Lambda^{Z_{AB}}_{(1-2\bar{e}_Z)^{2m-2}}$ on qubits AB. Since these channels are considered to be applied to a *bipartite* cluster state, as noted in Supplementary Fig. 4**b**, the effects of $\Lambda^{Z_B}_{1-2\bar{e}_X}$ and $\Lambda^{Z_{AB}}_{(1-2\bar{e}_Z)^{2m-2}}$ are equivalent to $\Lambda^{X_A}_{1-2\bar{e}_X}$ and $\Lambda^{Y_A}_{(1-2\bar{e}_Z)^{2m-2}}$, respectively.

Finally, we derive error probabilities of a Bell pair AB that is obtained by the success of our repeater protocol defined in Fig. 3. In the case of the success, the protocol can be regarded as a situation like the upper one of Supplementary Fig. 5 where the encoded complete-like cluster state $|\bar{G}^m_{\rm c}\rangle$ has already been transformed into a 4-qubit linear cluster state through the Z-basis measurements as in Supplementary Fig. 4**a**, and the 2nd-leaf qubits in the 4-qubit linear cluster state are connected via the successful Bell measurement defined in Fig. 2**a**. This situation can further be transformed into the the lower one of Supplementary Fig. 5, according to the rule of Supplementary Fig. 4**b**. Therefore, considering the depolarization $\mathcal{E}$ for the 2nd-leaf qubits, we conclude that the finally obtained entangled pair AB in a cluster state has errors specified by channel

$$\bar{\mathcal{E}}^{\rm tot}_A := (\Lambda^{Z_A}_{1-2\bar{e}_X})^n(\Lambda^{X_A}_{1-2\bar{e}_X})^n(\Lambda^{Y_A}_{(1-2\bar{e}_Z)^{2m-2}})^n\mathcal{E}^{2(n+1)}_A = \Lambda^{Z_A}_{(1-2\bar{e}_X)^n}\Lambda^{X_A}_{(1-2\bar{e}_X)^n}\Lambda^{Y_A}_{(1-2\bar{e}_Z)^{(2m-2)n}}\mathcal{E}^{2(n+1)}_A, \tag{37}$$

on average. If we rewrite $\bar{\mathcal{E}}^{\rm tot}_A$ in a standard form as

$$\bar{\mathcal{E}}^{\rm tot}_A(\hat{\rho}) = (1-\bar{E}_X-\bar{E}_Y-\bar{E}_Z)\hat{\rho} + \bar{E}_X\hat{X}_A\hat{\rho}\hat{X}_A + \bar{E}_Y\hat{Y}_A\hat{\rho}\hat{Y}_A + \bar{E}_Z\hat{Z}_A\hat{\rho}\hat{Z}_A, \tag{38}$$

the error probabilities are

$$\bar{E}_Z = \bar{E}_X = \frac{1}{4} - \frac{1}{4}(1-2e_{\rm m})^{2(n+1)}(1-2\bar{e}_X)^{2n}, \tag{39}$$

$$\bar{E}_Y = \frac{1}{4} + \frac{1}{4}(1-2e_{\rm m})^{2(n+1)}(1-2\bar{e}_X)^{2n} - \frac{1}{2}(1-2e_{\rm m})^{2(n+1)}(1-2\bar{e}_X)^{n}(1-2\bar{e}_Z)^{(2m-2)n}, \tag{40}$$

where we replace the error probability $e_{\rm d}$ of the depolarizing channel $\mathcal{E}$ with $e_{\rm m}$ by using Eq. (6). Then, note that the average fidelity F is given by

$$\bar{F} = 1 - \bar{E}_X - \bar{E}_Y - \bar{E}_Z. \tag{41}$$

Let us discuss the average errors $\bar{E}_Z$, $\bar{E}_X$ and $\bar{E}_Y$. The one-type-error channels $\Lambda^{Z_A}_{(1-2\bar{e}_X)^n}$, $\Lambda^{X_A}_{(1-2\bar{e}_X)^n}$ and $\Lambda^{Y_A}_{(1-2\bar{e}_Z)^{(2m-2)n}}$ in Eq. (37) come from transmission errors for single photons that compose the encoded 1st-leaf qubits. Actually, the effect of these channels to the final fidelity $\bar{F}$ is so small that $(1-2\bar{e}_Z)^{(2m-2)n} \simeq (1-2\bar{e}_X)^{2n} \simeq 1$ in Eqs. (39) and (40) are good approximation in reasonable overall distances L as will be seen in the next section, thanks to the special robustness of the loss-tolerant Z-basis or X-basis measurement against depolarization (as seen in numerical examples in Supplementary Note 1). Since *the 1st-leaf qubits of the state* $|\bar{G}^m_{\rm c}\rangle$ *correspond to quantum memories at a single repeater node in the conventional repeaters* [12–33] as described in the main body of the paper, this robustness of the encoded 1st-leaf qubits in our protocol is an answer to the problem on how to overcome practically time-dependent errors on quantum memories at repeater nodes in the conventional repeaters. This is in contrast to conventional repeater theories [12–20, 22, 23, 25–31] (except for Refs. [4, 21, 24, 32, 33]) that do not consider such *time-dependent* errors on their matter quantum memories, although they are the dominant noise for the matter quantum memories analogously to the loss for photons [39].

On the other hand, the depolarizing channel $\mathcal{E}^{2(n+1)}_A$ in Eq. (37) represents the effect of errors on the bare 2nd-leaf qubits. Since *the 2nd-leaf qubits serve as the single photons to supply entanglement to the 1st-leaf qubits (corresponding to quantum memories in the conventional quantum repeaters [12–33]) between adjacent repeater nodes* as noted in the main body of the paper, the effect of the errors must be shared by all the quantum repeater schemes [12–33] that rely on the entanglement generation by the transmission of bare single photons between adjacent repeater nodes. In principle, the errors could be overcome by invoking entanglement purification, which would be true even for our all photonic scheme if we could equip it with entanglement purification like a scheme [31] in a time-reversed fashion (c.f., Supplementary Discussion). However, in practice, the dominant noise of the optical channels is the loss, and the channel errors described by a depolarizing channel $\mathcal{E}$ are incomparably smaller than that [39]. Thus, $(1-4e_{\rm d}/3)^{2(n+1)} = (1-2e_{\rm m})^{2(n+1)} \simeq 1-4(n+1)e_{\rm m}$ in Eqs. (39) and (40) are good approximation, and entanglement purification to overcome the errors induced by the depolarizing channel $\mathcal{E}^{2(n+1)}_A$ has conventionally been considered [13, 20] to be unnecessary in *reasonable* communication distances L.

Therefore, as long as we are interested in quantum communication over reasonable communication distances L, the average errors are approximated to be

$$\bar{E}_Z = \bar{E}_X \simeq \bar{E}_Y \simeq \frac{1}{4} - \frac{1}{4}(1-2e_{\rm m})^{2(n+1)} \simeq (n+1)e_{\rm m} = (n+1)\frac{2e_{\rm d}}{3} = \frac{2e_{\rm d}}{3}\frac{L}{L_0}. \tag{42}$$

Thus, from Eq. (41), the average fidelity $\bar{F}$ of the final entangled pair is approximately

$$\bar{F} = 1 - \bar{E}_Z - \bar{E}_X - \bar{E}_Y \simeq 1 - 3e_{\mathrm{m}}\frac{L}{L_0} = 1 - 2e_{\mathrm{d}}\frac{L}{L_0}. \tag{43}$$

The validity of these approximations will be confirmed in the next section by numerical examples based on the exact formulas (39)-(41).

Numerical examples. To show the polynomial scaling of our protocol explicitly, we first estimate $\bar{Q}$, P, $\bar{R}$, $\bar{E}_Z$, $\bar{E}_X$, $\bar{E}_Y$ and $\bar{F}$ for four cases. Then, we provide a comparison between our protocol and the speediest protocol [4], referencing the basic assumptions made in the updated version [42] of Ref. [4].

Let us assume that photons always run in optical fibres with the transmittance $T = e^{-l/l_{\mathrm{att}}}$ for distance l ($l_{\mathrm{att}} =$ 22 km) from the birth toward the generation process for $|\bar{G}_{\mathrm{c}}^{m}\rangle$. In addition, we suppose that the optical fibres have small errors when they are used to connect distant repeater stations ($L_0/2$ apart), and that the errors of the fibre with length $L_0/2$ can be described as an individual depolarizing channel with error probability e_{d}. We also assume to use single photon sources with efficiency η_{S} and photon detectors with quantum efficiency η_{D}. In the estimation of ϵ_0 of Eq. (28), by assuming that every step in our protocol including the preparation is executed in the optical fibre, we use $P_{\tau_{\mathrm{a}}} = e^{-c\tau_{\mathrm{a}}/l_{\mathrm{att}}}$ with $c = 2 \times 10^8$ m s^{-1}. Suppose that the repetition rate f of the slowest device among single-photon sources, photon detectors and active feedforward techniques is $f = 100$ kHz [40]. Then, by combining the numerical results (I) and (II) obtained in Supplementary Note 1 and Eqs. (19), (22), (23)-(26) and (39)-(41), we obtain the following results under numerical calculation *to minimize* $\bar{Q}$: (I) For $L = 5000$ km ($L = 1000$ km), $L_0 = 4$ km, $e_{\mathrm{m}} = 2e_{\mathrm{d}}/3 = 2.8 \times 10^{-5}$, $\eta_{\mathrm{D}}\eta_{\mathrm{S}} = 0.95$ and $\tau_{\mathrm{a}} = 150$ ns, by choosing $m = 24$ ($m = 19$) and $\{b_0, b_1, b_2\} = \{16, 14, 1\}$ ($\{b_0, b_1, b_2\} = \{11, 11, 1\}$), we obtain $\epsilon_0 \simeq 0.20$ and $\tau_{\mathrm{c}} = 3.1$ μs ($\tau_{\mathrm{c}} = 2.9$ μs), which presents $\bar{Q} = 4.0 \times 10^7$ ($\bar{Q} = 4.1 \times 10^6$), $P = 0.69$ ($P = 0.58$), $\bar{R} = 69$ kHz ($\bar{R} = 58$ kHz), $\bar{E}_Z = \bar{E}_X = 3.5 \times 10^{-2}$ ($\bar{E}_Z = \bar{E}_X = 8.9 \times 10^{-3}$), $\bar{E}_Y = 3.3 \times 10^{-2}$ ($\bar{E}_Y = 7.6 \times 10^{-3}$) and $\bar{F} = 0.90$ ($\bar{F} = 0.97$). This choice of the parameters allows us to achieve $F \geq 0.9$ as in the analysis of conventional quantum repeater schemes [20]. (II) For $L = 5000$ km ($L = 1000$ km), $L_0 = 8$ km, $e_{\mathrm{m}} = 2e_{\mathrm{d}}/3 = 5.6 \times 10^{-5}$, $\eta_{\mathrm{D}}\eta_{\mathrm{S}} = 0.95$ and $\tau_{\mathrm{a}} = 150$ ns, by choosing $m = 27$ ($m = 21$) and $\{b_0, b_1, b_2\} = \{17, 28, 2\}$ ($\{b_0, b_1, b_2\} = \{12, 23, 2\}$), we obtain $\epsilon_0 \simeq 0.27$ and $\tau_{\mathrm{c}} = 3.4$ μs ($\tau_{\mathrm{c}} = 3.2$ μs), which presents $\bar{Q} = 7.6 \times 10^7$ ($\bar{Q} = 7.3 \times 10^6$), $P = 0.65$ ($P = 0.60$), $\bar{R} = 65$ kHz ($\bar{R} = 60$ kHz), $\bar{E}_Z = \bar{E}_X = 4.0 \times 10^{-2}$ ($\bar{E}_Z = \bar{E}_X = 1.2 \times 10^{-2}$), $\bar{E}_Y = 3.3 \times 10^{-2}$ ($\bar{E}_Y = 7.6 \times 10^{-3}$) and $\bar{F} = 0.89$ ($\bar{F} = 0.97$). Here we assumed that the errors of the fibre with 4 km in the case (II) can be described as a series of two depolarizing channels for 2-kilometre fibre in the case (I), which is valid as long as the overall transmittance $1 - \epsilon_0$ for photons is larger than the dark count probability of photon detectors [41] (this is indeed the case for our repeater settings).

Comparison with the speediest protocol given by Munro et al. [4].—Let us compare our protocol with the speediest protocol given by Munro *et al.* [4]. Here we refer to the data in the up-to-date version [42] of Ref. [4].

Munro *et al.*'s scheme uses single-photon sources and photon detectors, similarly to our proposal. Thus, in both of the protocols, suppose that the single-photon source has 97% efficiency and the photon detector has 97% quantum efficiency, following Ref. [42]. In addition, we assume that the photon detector is the slowest device with 100 kHz repetition rate,. i.e., $f = 100$ kHz. We also assume that the attenuation length of the fibre is 22 km, and 0.1% general errors occur in the transmission of the fibre with 10 km according to Ref. [42]. Although Munro *et al.* sometimes in their paper [42] assume that a single photon corresponds to multiple qubits (by using the multiple degrees of freedom such as time bin, polarization and spatial modes), this assumption can be shared even with our protocol [43] to decrease the required photon number, and it is thus irrelevant when we make a comparison between our protocol and Munro *et al.*'s protocol. Therefore, for fairness and simplicity, here we conservatively consider that a single photon simply corresponds to a single qubit in both of the protocols. Let us consider 800 km quantum communication ($L = 800$ km), according to the setting of Ref. [42].

Performance of Munro *et al.*'s scheme: Suppose that the coupling between a single photon and a matter qubit is assumed to be 97% [42]. Since Table 1 of Ref. [42] has values until $p = 0.67$, we choose the distance between adjacent nodes $L_0 = 6.15$ km (i.e., 129 repeater nodes), because the choice actually presents $p = 0.97^4 \times e^{-6.15/22} = 0.67$. We assume that the fibre with 6.15 km includes 0.0615%[$= 0.1\% \times (6.15\ \mathrm{km}/10\ \mathrm{km})$]. Then, the Table 1 under one qubit/photon shows that the number of the matter quantum memories at each node is $13 \times 1500 = 19500$. Since quantum information of a single matter quantum memory is exchanged with a single photon, the number of single photons prepared at each node is also 19500. Thus, the total number of the consumed photons for each trial is $19500 \times (129 + 1) = 2.5 \times 10^6$, and the total number of matter quantum memories in repeater nodes is $19500 \times 129 = 2.5 \times 10^6$. Since the protocol almost deterministically generates the entanglement pair with approximate fidelity 92%[$= 100\% - (129 + 1) \times 0.0615\%$] [43] for each trial, the rate

to produce the entangled pair is about 100 kHz according to the repetition rate of the single-photon detectors, i.e., for $f = 100$ kHz.

Performance of our scheme: Suppose that the distance between adjacent source repeater nodes is similarly $L_0 =$ 6.15 km (i.e., 129 source repeater nodes). This choice implies the use of fibres with (6.15/2) km, which have 0.0308%[= 0.1% × (6.15 km/2)/10 km)] errors (i.e., $e_{\rm d} = 0.0308$). By choosing $m = 20$ and $\{b_0, b_1, b_2\} = \{10, 20, 2\}$, we obtain $\epsilon_0 \simeq 0.25$ and $\tau_{\rm c} = 3.1$ μs, which presents $\bar{Q} = 5.3 \times 10^6$, $P = 0.60$, $\bar{R} = 60$ kHz, $\bar{E}_Z = \bar{E}_X = 4.1\%$, $\bar{E}_Y = 2.9\%$ and $\bar{F} = 89\%$, where the number of photons prepared at each node for each trial is 24440. Thus, the average total number of the photons consumed until producing the entangled pair with 89% fidelity is 5.3×10^6, and the rate to produce the entangled pair is 60 kHz.

According to this comparison, our protocol is comparable with the speediest protocol of Munro *et al.* in the entanglement generation rate, although Munro *et al.*'s protocol uses not only single photons but also demanding matter quantum memories and both of their required numbers are in the same order of the consumed photons in our protocol.

Supplementary Discussion

As shown in the error analysis for our repeater protocol and the numerical examples in Supplementary Note 3, the average fidelity $\bar{F}$ of the final entangled pair is degraded almost only by the small channel errors on the (bare) 2nd-leaf qubits contributing to the final pair. Since the number of the 2nd-leaf qubits increases with overall distance L, the final fidelity degrades with L. However, this degradation could be overcome by equipping our protocol with entanglement purification or quantum error correction. In fact, there is a possibility that even two-way entanglement purification can be installed in our protocol. Let us discuss this point first.

We first note that our protocol has not yet utilized all the successfully connected arms of the complete-like cluster states $|\bar{G}_{\rm c}^m\rangle^{\otimes 2}$—which could be made at step (ii) of our protocol in Fig. 3—at a single receiver node, although six or more successful arms would exist on average from the numbers m in the numerical examples in Supplementary Note 3. These arms, which are entanglement between the 1st-leaf qubits, are analogous to the existence of plural entangled pairs between adjacent repeater nodes in the conventional quantum repeaters. Therefore, we can naturally expect the existence of a mechanism to purify entanglement between them. Moreover, this entanglement purification can be made *locally* for our protocol, because the connected 1st-leaf qubits exist at a *single* receiver node in contrast to conventional entanglement purification which is executed between *distant* repeater nodes by exchanging the heralding signals with the help of quantum memories. This distinguished feature of our all photonic protocol may allow even time-reversed two-way entanglement purification of Ref. [31] to be made locally and without quantum memories. Therefore, our scheme could be modified such that it incorporates *any* entanglement purification *at least between adjacent repeater nodes* in a time-reversed fashion. More generally, our scheme is compatible with entanglement purification or error-detection/correction protocols if one employs a one-way protocol for entanglement purification or standard error-detection/correction protocols or decoherence-free subspace ideas.

In contrast, it is an open problem whether the *two-way* entanglement purification between repeater nodes *distant beyond neighbouring*—which is needed to perform the nested purification protocol [12]—will work in our method or indeed arbitrary quantum repeater schemes with matter quantum memories under practical situations. In practice [39], the photons and matter quantum memories inevitably receive loss and depolarization/dephasing *exponentially with time*, respectively. The exponential scaling of the noises was not considered in the earliest literature [12]. But, the scaling throws a doubt on the key assumption in Ref. [12] that the performance of the two-way entanglement purification protocol is determined only by the local devices (i.e., independently of the distance), because the protocol indeed, due to the probabilistic nature, needs the waiting time for the classical communication between the long distant repeater nodes and such long waiting time leads to the exponential noises. This could change the scaling of the nested purification protocol [12] from the very similar reasoning of Razavi *et al.* [21] which changed the scaling of the quantum repeaters with atomic-ensemble quantum memories [13, 20] by taking into account the effect of the finiteness of the coherence time. Therefore, it is open whether we can incorporate such two-way entanglement purification in our scheme as well as any other quantum repeater schemes with retaining its advantage. The detailed investigation is very interesting but outside the scope of the present paper. Fortunately, as noted earlier in the error analysis for our repeater protocol in Supplementary Note 3, entanglement purification protocols have conventionally been considered [13, 20] to be unnecessary in reasonable communication distances L that are needed on the Earth. Hence, from a practical standpoint, the practicality of our scheme is not seriously affected by those future investigations on the feasibility of combining two-way entanglement purification protocols with our scheme (and indeed many other

quantum repeater schemes).

Supplementary References

[1] Varnava, M., Browne, D. E. & Rudolph, T. Loss tolerance in one-way quantum computation via counterfactual error correction. *Phys. Rev. Lett.* **97**, 120501 (2006).
[2] Varnava, M., Browne, D. E. & Rudolph, T. How good must single photon sources and detectors be for efficient linear optical quantum computation? *Phys. Rev. Lett.* **100**, 060502 (2008).
[3] Varnava, M., Browne, D. E. & Rudolph, T. Loss tolerant linear optical quantum memory by measurement-based quantum computing. *New J. Phys.* **9**, 203 (2007).
[4] Munro, W. J., Stephens, A. M., Devitt, S. J., Harrison, K. A. & Nemoto, K. Quantum communication without the necessity of quantum memories. *Nature Photon.* **6**, 777-781 (2012).
[5] Gottesman, D. The Heisenberg representation of quantum computers. *In Group22: Proceedings of the XXII International Colloquium on Group Theoretical Methods in Physics*, 32-43 (Cambridge, MA, International Press, 1999).
[6] Raussendorf, R. & Briegel, H. J. A one-way quantum computer. *Phys. Rev. Lett.* **86**, 5188-5191 (2000).
[7] Hein, M. *et al.* Entanglement in graph states and its applications. Preprint at ⟨http://arxiv.org/abs/quant-ph/0602096⟩.
[8] Chen, Q., Cheng, J., Wang, K.-L. & Du, J. Efficient construction of two-dimensional cluster states with probabilistic quantum gates. *Phys. Rev.* A **73**, 012303 (2006).
[9] Fujii, K. & Tokunaga, Y. Fault-tolerant topological one-way quantum computation with probabilistic two-qubit gates *Phys. Rev. Lett.* **105**, 250503 (2010).
[10] Prevedel, R. *et al.* High-speed linear optics quantum computing using active feed-forward. *Nature* **445**, 65-69 (2007).
[11] Bennett, C. H. *et al.* Teleporting an unknown quantum state via dual classical and Einstein-Podolsky-Rosen channels. *Phys. Rev. Lett.* **70**, 1895-1898 (1993).
[12] Briegel, H. J., Dür, W., Cirac, J. I. & Zoller, P. Quantum repeaters: The role of imperfect local operations in quantum communication. *Phys. Rev. Lett.* **81**, 5932-5935 (1998).
[13] Duan, L.-M., Lukin, M. D., Cirac, J. I. & Zoller, P. Long-distance quantum communication with atomic ensembles and linear optics. *Nature* **414**, 413-418 (2001).
[14] Kok, P., Williams, C. P. & Dowling, J. P. Construction of a quantum repeater with linear optics. *Phys. Rev. A* **68**, 022301 (2003).
[15] Childress, L., Taylor, J. M., Sørensen, A. S. & Lukin, M. D. Fault-tolerant quantum communication based on solid-state photon emitters. *Phys. Rev. Lett.* **96**, 070504 (2006).
[16] van Loock, P. *et al.* Hybrid quantum repeater using bright coherent light. *Phys. Rev. Lett.* **96**, 240501 (2006).
[17] Ladd, T. D. *et al.* Hybrid quantum repeater based on dispersive CQED interactions between matter qubits and bright coherent light. *New J. Phys.* **8**, 184 (2006).
[18] Simon, C. *et al.* Quantum repeaters with photon pair sources and multimode memories. *Phys. Rev. Lett.* **98**, 190503 (2007).
[19] Kimble, H. J. The quantum internet. *Nature* **453**, 1023-1030 (2008).
[20] Sangouard, N., Simon, C., de Riedmatten, N. & Gisin, N. Quantum repeaters based on atomic ensembles and linear optics. *Rev. Mod. Phys.* **83**, 33-80 (2011).
[21] Razavi, M., Piani, M. & Lütkenhaus, N. Quantum repeaters with imperfect memories: Cost and scalability. *Phys. Rev. A* **80**, 032301 (2009).
[22] van Loock, P., Lütkenhaus, N., Munro, W. J. & Nemoto, K. Quantum repeaters using coherent-state communication. *Phys. Rev. A* **78**, 062319 (2008).
[23] Munro, W. J., Van Meter, R., Louis, S. G. R. & Nemoto, K. High-bandwidth hybrid quantum repeater. *Phys. Rev. Lett.* **101**, 040502 (2008).
[24] Jiang, L. *et al.* Quantum repeater with encoding. *Phys. Rev.* A **79**, 032325 (2009).
[25] Azuma, K. *et al.* Optimal entanglement generation for efficient hybrid quantum repeaters. *Phys. Rev. A* **80**, 060303(R) (2009).
[26] Azuma, K., Sota, N., Koashi, M. & Imoto, N. Tight bound on coherent-state-based entanglement generation over lossy channels *Phys. Rev. A* **81**, 022325 (2010).
[27] Azuma, K., Takeda, H., Koashi, M. & Imoto, N. Quantum repeaters and computation by a single module: Remote nondestructive parity measurement. *Phys. Rev. A* **85**, 062309 (2012).
[28] Wang, T.-J., Song, S.-Y. & Long, G. L. Quantum repeater based on spatial entanglement of photons and quantum-dot spins in optical microcavities. *Phys. Rev. A* **85**, 062311 (2012).
[29] Azuma, K. & Kato, G. Optimal entanglement manipulation via coherent-state transmission. *Phys. Rev. A* **85**, 060303(R) (2012).
[30] Li, Y., Barrett, S. D., Stace, T. M. & Benjamin, S. C. Long range failure-tolerant entanglement distribution. *New J. Phys.* **15**, 023012 (2013).
[31] Zwerger, M., Dür, W. & Briegel, H. J. Measurement-based quantum repeaters. *Phys. Rev. A* **85**, 062326 (2012).
[32] Munro, W. J., Harrison, K. A., Stephens, A. M., Devitt, S. J. & Nemoto, K. From quantum multiplexing to high-

performance quantum networking. *Nature Photon.* **4**, 792-796 (2010).
[33] Grudka, A. *et al.* Long-distance quantum communication over noisy networks without long-time quantum memory. *Phys. Rev. A* **90**, 062311 (2014).
[34] Vaidman, L. Instantaneous measurement of nonlocal variables. *Phys. Rev. Lett.* **90**, 010402 (2003).
[35] Clark, S. R., Connor, A. J., Jaksch, D. & Popescu, S. Entanglement consumption of instantaneous nonlocal quantum measurements. *New J. Phys.* **12**, 083034 (2010).
[36] Lau, H.-K. & Lo, H.-K. Insecurity of position-based quantum-cryptography protocols against entanglement attacks. *Phys. Rev. A* **83**, 012322 (2011).
[37] Kent, A., Munro, W. J. & Spiller, T. P. Quantum tagging: Authenticating location via quantum information and relativistic signaling constraints. *Phys. Rev. A* **84**, 012326 (2011).
[38] Buhrman, H. *et al.* Position-based quantum cryptography: Impossibility and constructions. *Advances in Cryptology – CRYPTO 2011* **6841**, 429-446 (Springer Berlin Heidelberg, 2011).
[39] Ladd, T. D. *et al.* Quantum computers. *Nature* **464**, 45 (2010).
[40] Hadfield, R. H. Single-photon detectors for optical quantum information applications. *Nature Photon.* **3**, 696-705 (2009).
[41] Brassard, G., Lütkenhaus, N., Mor, T. & Sanders, B. C. Limitations on practical quantum cryptography. *Phys. Rev. Lett.* **85**, 1330-1333 (2000).
[42] Munro, W. J., Stephens, A. M., Devitt, S. J., Harrison, K. A. & Nemoto, K. Quantum communication without the necessity of quantum memories. Preprint at ⟨http://arxiv.org/abs/1306.4137⟩.
[43] Private communication with W. J. Munro.
